# Crystal chemistry of layered structures formed by linear rigid silyl-capped molecules

**DOI:** 10.1107/S2052252515011665

**Published:** 2015-08-20

**Authors:** Daniel Lumpi, Paul Kautny, Berthold Stöger, Johannes Fröhlich

**Affiliations:** a Institute of Applied Synthetic Chemistry, Vienna University of Technology, Vienna, Austria; b Institute of Chemical Technologies and Analytics, Vienna University of Technology, Vienna, Austria

**Keywords:** arene spacers, incommensurately modulated structures, layer interfaces, order–disorder polytypes, Hirshfeld surface fingerprint plots

## Abstract

The crystallization behavior of methylthio- or methylsulfonyl-containing spacer extended *Z*,*Z*-*bis*-ene–yne molecules capped with trimethylsilyl groups obtained by (tandem) thiophene ring fragmentation and of two non-spacer extended analogs were investigated. The rigid and linear molecules generally crystallized in layers whereby the flexibility of the layer interfaces formed by the silyl groups leads to a remarkably rich crystal chemistry. The molecules with benzene and thiophene spacers both crystallized with *C*2/*c* symmetry and can be considered as merotypes. Increasing the steric bulk of the core by introduction of ethylenedioxythiophene (EDOT) gave a structure incommensurately modulated in the [010] direction. Further increase of steric demand in the case of a dimethoxythiophene restored periodicity along [010] but resulted in a doubling of the **c** vector. Two different polytypes were observed, which feature geometrically different layer interfaces (non-OD, order–disorder, polytypes), one with a high stacking fault probability. Oxidation of the methylthio groups of the benzene-based molecule to methylsulfonyl groups led to three polymorphs (two temperature-dependent), which were analyzed by Hirshfeld surface *d*_e_/*d*_i_ fingerprint plots. The analogously oxidized EDOT-based molecule crystallized as systematic twins owing to its OD polytypism. Shortening of the backbone by removal of the aryl core resulted in an enantiomorphic structure and a further shortening by removal of a methylthio-ene fragment again in a systematically twinned OD polytype.

## Abstract 2

Silyl capped aryl *bis*-ene–yne compounds and their derivatives possess a rich crystal chemistry: merotypism, polymorphism, polytypism, twinning and incommensurate modulation.

## 1. Introduction   

The controlled formation of layers plays an important role in the design of materials. For example, hybrid organic–inorganic layered perovskites (Mitzi, 2001 ▸) are natural quantum well structures and can be tuned to specific electronic, magnetic and optical properties. The combination of layers with different properties enables the synthesis of multi-functional composites (Coronado *et al.*, 2000 ▸). In the field of organic electronics the formation of layers has been shown to be a viable strategy to improve conductivity (Anthony *et al.*, 2001 ▸). Efficient charge transport in organic materials is governed by nearest-neighbor electronic coupling. The intermolecular coupling is maximized when a face-to-face orientation of aromatic molecules is realized, as a consequence of enhanced interactions of the π-electron clouds of adjacent molecules (Mueller & Bunz, 2007 ▸).

**Figure 1:**
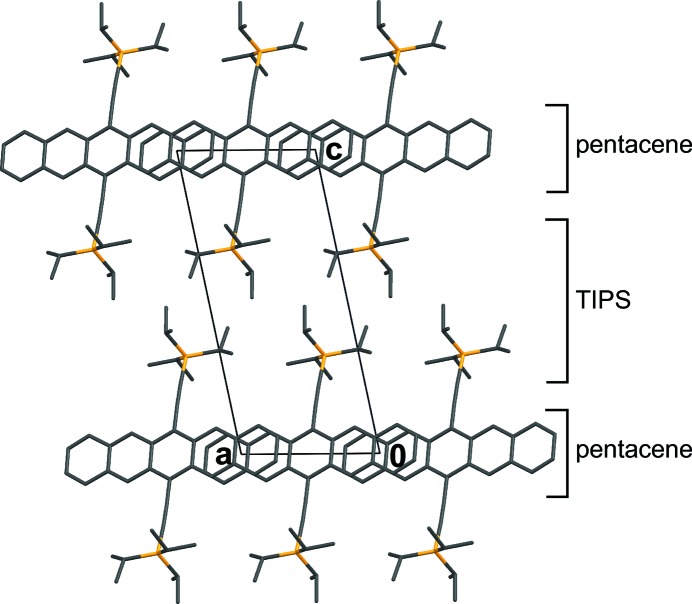
Crystal structure of TIPS-pentacene (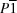) featuring distinct layers of pentacene cores and TIPS groups connected by —C C—bridges viewed down [010]. Coordinates taken from Anthony *et al.* (2001 ▸). H atoms and disorder of the TIPS groups were omitted for clarity.

Anthony *et al.* (2001 ▸) modified pentacene by connecting it at the central C atoms to triisopropylsilyl (TIPS) groups *via* rigid ethyne bridges. As opposed to plain pentacene, the resulting TIPS-pentacene molecules crystallized in layers (Fig. 1 ▸), whereby the pentacene cores are arranged in face-to-face orientation. As a result, a significantly lower resistivity perpendicular to the pentacene was reported (Anthony *et al.*, 2001 ▸). Hence, TIPS-pentacene yielded promising solution-processed OFET (organic field effect transistor) devices (Park *et al.*, 2007 ▸). In addition to the modified stacking arrangement the structures showed improved stability and solubility. On this basis a general molecular design for improved π-stacking was proposed by Anthony *et al.* (2002 ▸), and intense research in the field of substituted acene derivatives arose from these findings and is of ongoing interest (Anthony, 1994 ▸, 2008 ▸).

Besides technological importance, layered structures are interesting from a crystallographic point of view. Polymorphs (different crystal structures of the same composition) that crystallize in different arrangements of equivalent layers are called polytypes. Polytypes are ubiquitous in all classes of solid materials and are often the cause of crystallographically challenging problems, like twinning (the systematic association of equivalent macroscopic domains with different orientations; Hahn, 2006*b* ▸) and stacking disorder. In order–disorder (OD) polytyes (Dornberger-Schiff & Grell-Niemann, 1961 ▸; Ferraris *et al.*, 2008 ▸) pairs of layers are equivalent and therefore all polytypes are locally equivalent. The symmetry of a polytype is described by groupoids, a generalization of the group concept (Fichtner, 1986 ▸). For OD polytypes these groupoids are classified into OD groupoid families (Fichtner, 1977*b* ▸), in analogy to space group types. These were tabulated for the special case of OD structures composed of layers of one kind with identical lattices (Fichtner, 1977*a* ▸).

Nevertheless, many issues remain unsolved. For example, OD structures composed of layers with different lattices have received only a little attention. Yet in some structures, like K_2_HAsO_4_·2.5H_2_O (Stöger, Weil & Zobetz, 2012 ▸), the different lattices of the layers are the decisive factor giving rise to OD polytypism. Moreover, we have discovered structures that follow the basic principle of OD theory, namely locally equivalent stacking possibilities, but do not follow the strict definition of OD theory (Stöger, Kautny *et al.*, 2012 ▸; Stöger & Weil, 2013 ▸). Also, as we will show in this work, polytypes that are not locally equivalent must not be overlooked. The symmetry groupoids of these kinds of polytypes are virtually unexplored. Thus, to shed new light on OD theory and related kinds of polytypism, we are in search of suitable model compounds. A fundamental advantage of organic over in­organic compounds is the ease of introduction of systematic geometric and electronic modifications.

**Figure 2:**
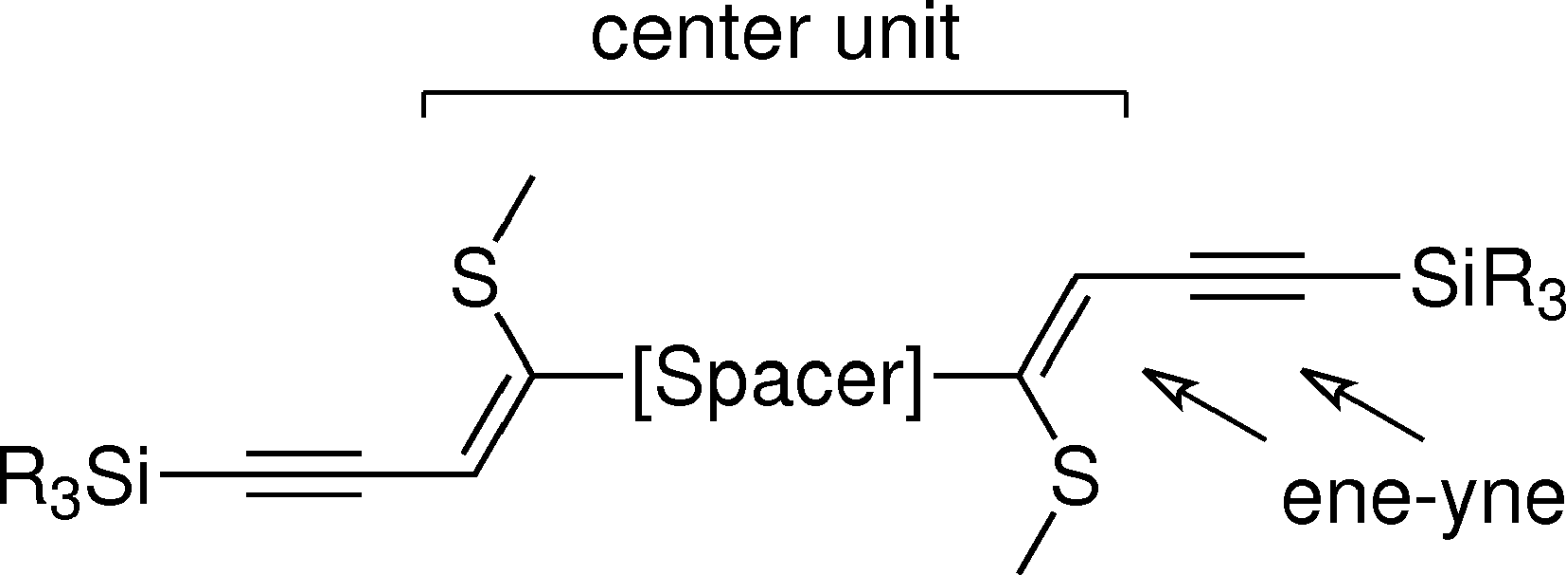
General structure of spacer-extended ene–yne molecules.

An ideal basis for the crystal engineering of layered structures seemed to be spacer-extended ene–yne molecules synthesized by tandem thiophene ring-fragmentation (TRF) reactions (Bobrovsky *et al.*, 2008 ▸) owing to the generality and flexibility of the TRF protocol. The makeup of these mol­ecules resembles the TIPS-pentacene described above, though with smaller aromatic rings and a side chain extended by an ene fragment and a methylthio group (Fig. 2 ▸). An interesting aspect of these ene–yne scaffolds is the possibility to selectively oxidize the methylthio group to modify electronic properties, but also introduce structure-directing hydrogen-bond acceptors.

The first reports on controlled TRF reactions go back to Gronowitz & Torbjörn (1970 ▸) and Jakobsen (1970 ▸). They were explored in detail by Gronowitz & Frejd (1978 ▸), Iddon (1983 ▸) and Gilchrist (1987 ▸). This approach enables the selective yield of *Z*-isomers of ene–yne compounds as determined by the cyclic structure of thiophene. The exploration of tandem fragmentation reactions affording double-sided ring-opened (ene–yne) products was first reported by Fuller *et al.* (1999 ▸) on substituted thieno[3,2-*b*]thiophenes.

The first synthesized molecule of the class depicted in Fig. 2 ▸ was BSEM (**1**) [benzene spacer-extended with methylthio group; spacer = benzene, Si*R*_3_ = trimethylsilyl (TMS)]. As expected, in analogy to TIPS-pentacene and related molecules (Anthony *et al.*, 2001 ▸, 2002 ▸), BSEM (**1**) crystallizes in distinct molecular layers delimited by the bulky and flexible silyl groups. Therefore, this molecule was chosen as the starting point of the systematic crystallographic studies presented in this work.

It has to be noted that, in traditional crystal engineering, directed intermolecular interactions, notably *via* hydrogen bonding or, less commonly, halogen bonds, are used to induce controlled ‘self-assembly’ of molecules (Aakeröy *et al.*, 2010 ▸). In the case at hand, no such bonding exists, since the layer-delimiting moieties are trialkylsilyl groups. Nevertheless, the special makeup of the molecules clearly induces crystallization as layered structures, and therefore variations of the spacer-extended ene–yne compounds can be considered as a form of crystal engineering.

## 2. Results and discussion   

### 2.1. Molecular modifications   

**Figure 3:**
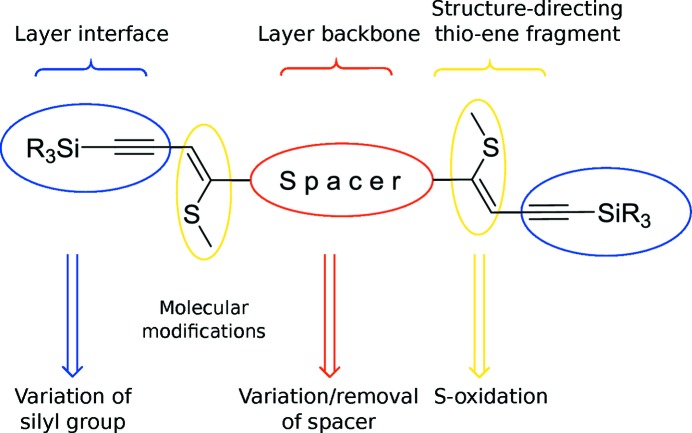
Scope of molecular modifications (bottom) and expected impact on the structures (top).

The scope of molecular modifications and expected impacts on the layer structures are schematized in Fig. 3 ▸. The main focus is modification of the spacer to control the intermolecular spacing and width of the layer backbone, with special attention paid to the effects on the layer interface. The latter is also determined by the nature of the silyl groups. Finally, the possibility of introducing potential structure-directing hydrogen-bond acceptors by oxidation of the methylthio groups is used to create new kinds of layer structures.

#### 2.1.1. Spacer modifications   

**Figure 4:**
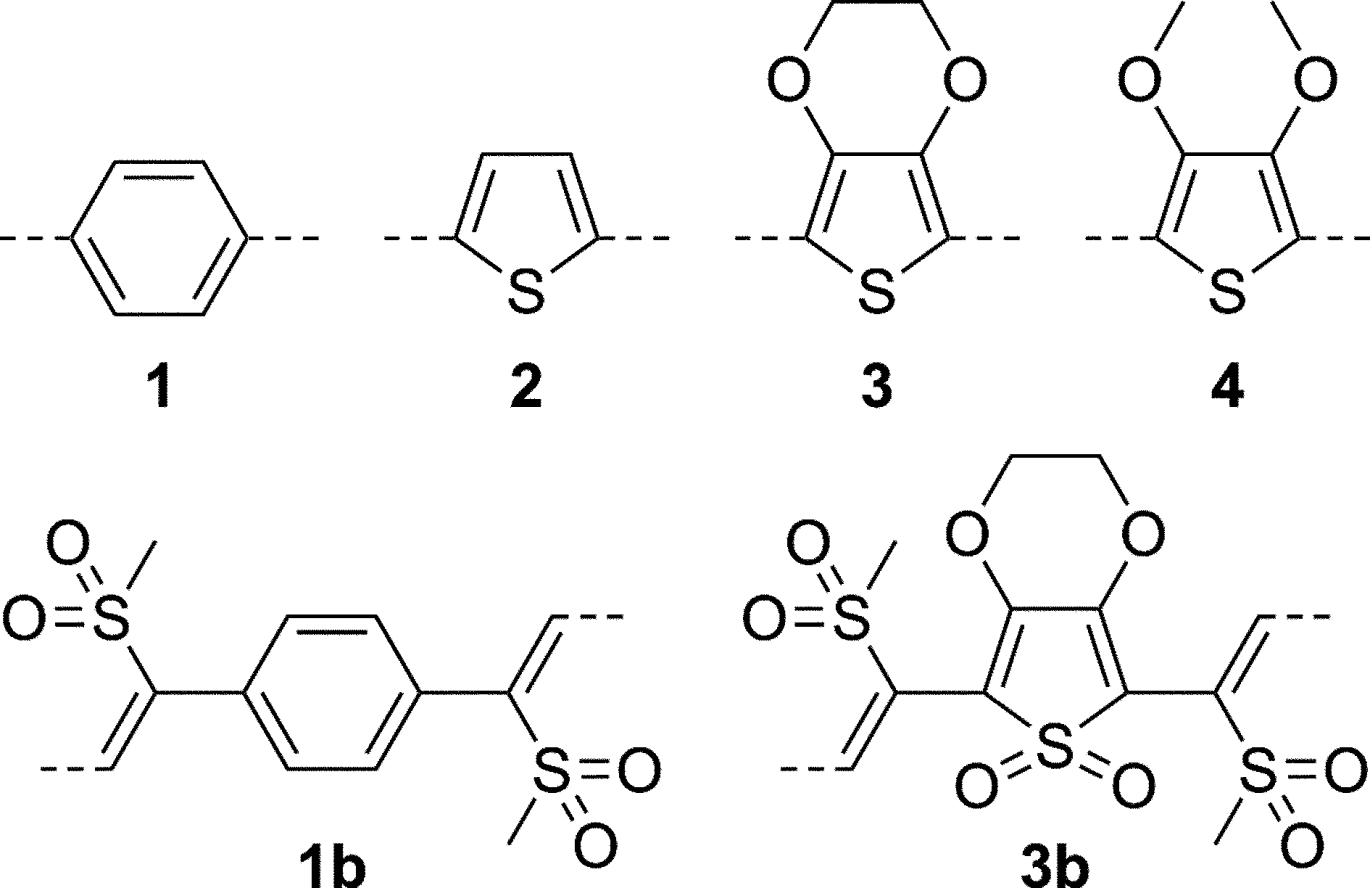
Spacer-extended ene–yne molecules.

The variations of the spacer unit are illustrated in Fig. 4 ▸. Firstly, the *para*-substituted benzene (*mmm*) spacer was replaced by the electron-rich 2,5-substituted thiophene (2*mm*) to TSEM (**2**). Thiophene is, from a technological point of view, an interesting core, since polythiophene has been successfully applied in the field of organic semiconductors.

To analyze the effects of a bulkier spacer extending into the layer plane, we enlarged the spacer to a 3,4-ethyl­ene­dioxythiophene (EDOT) bicycle, which is, like thiophene, commonly used in the field of organic semiconductors, to give ESEM (**3**). Surprisingly, the resulting crystal structure was incommensurately modulated. Such a structure can be described by a periodic basic structure and periodic modulation functions, but since the periodicities are incommensurate, the overall structure is only quasi-periodic (Janssen *et al.*, 2007 ▸; van Smaalen, 2007 ▸). A review of incommensurately modulated organic molecular structures was given by Schönleber (2011 ▸).

To better understand the reasons for the modulation, we synthesized the ring-opened 3,4-dimethoxythiophene compound DSEM (**4**) featuring even more steric bulk. After numerous failed crystallization attempts, we were able to obtain two non-incommensurate polymorphs, which can be considered as polytypes, from the same crystallization dish and which will be designated as polytype I and II, respectively.

#### 2.1.2. Oxidation to sulfonyl compounds   

The electronic makeup of the molecules was modified by oxidation of the thioether functionality to the corresponding sulfonyl functions (Lumpi *et al.*, 2014 ▸). We obtained crystals of the disulfonyl analog of BSEM (**1**): oxBSEM (**1*b***) and the fully oxidized trisulfonyl analog of ESEM (**3**): oxESEM (**3*b***).

For oxBSEM (**1*b***) we observed three polymorphs: polymorph I reversibly transforms into polymorph II upon cooling below *ca* 150 K. Polymorph III is unrelated to the former two and features no phase transition from 100 K up to the melting point. So far we were unable to determine the crystallization conditions needed to selectively obtain either polymorph.

#### 2.1.3. Backbone modifications   

**Figure 5:**
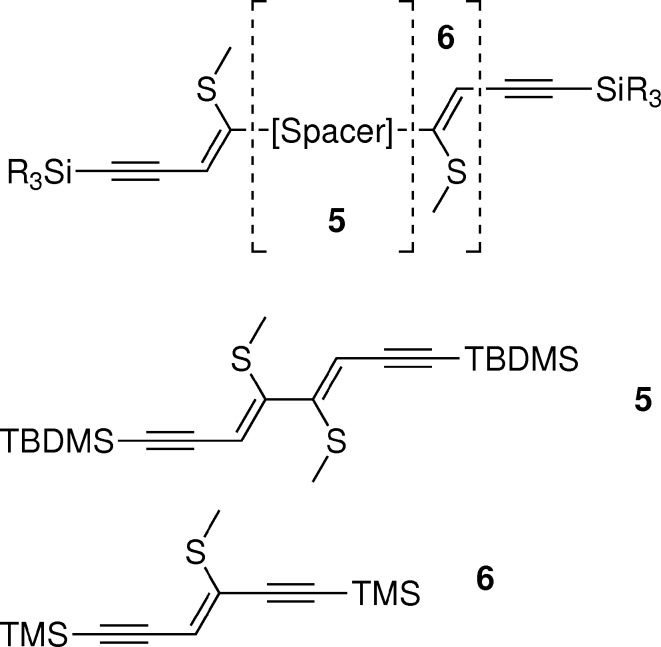
Non-spacer-extended ene–yne molecules.

An important argument of OD theory concerns the layer thickness: only for thick layers can interatomic interactions over a layer width be ignored. Thus, in a further modification we shortened the backbone (Fig. 5 ▸). At first the spacer was removed to NSEM (non-spacer-extended with methylthio group; Bobrovsky *et al.*, 2008 ▸). We were unable to obtain single crystals suitable for structure determination of the TMS-containing molecule. Therefore, we synthesized and grew crystals of the corresponding *tert*-butyl-dimethylsilyl (TBDMS) compound NSEM-TBDMS (**5**).

Finally, we shortened the backbone further by removing a —CH=C(SMe)— fragment to the single-sided ring-opened product ASYM (**6**). The name ASYM indicates a lack of symmetry in the direction of the main axis of the molecule. Despite the name, the molecule does not possess a stereogenic center and it can be considered as symmetric by reflection. Besides the short length, the compound seemed especially interesting in the light of OD theory, since the latter differentiates between polar and nonpolar layers. By growing crystals of a molecule that is polar with respect to the main axis, we were hoping to obtain polar layers, yet even ASYM (**6**) crystallized in nonpolar layers.

### 2.2. Molecular structures   

**Figure 6:**
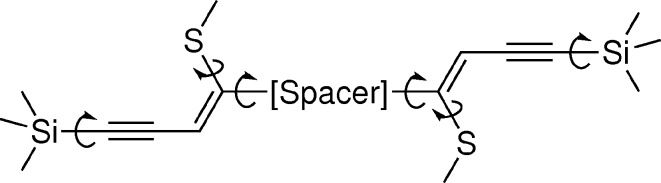
Rotational degrees of freedom in spacer-extended molecule.

The molecules presented in this work are essentially rigid, but possess three kinds of pivotal points, as depicted in Fig. 6 ▸. The main pivotal point is the connection of the side chains to the aromatic spacers (or the connection of the side chains in the case of non-spacer-extended molecules). Moreover, the methylthio (or methylsulfonyl) groups as well as the silyl groups can freely rotate. Nevertheless, the overall forms of the molecules feature little possibility for variation.

In Table 1 ▸ the rotation angles about the freely rotatable bonds of the title compounds are compiled.

**Figure 7:**
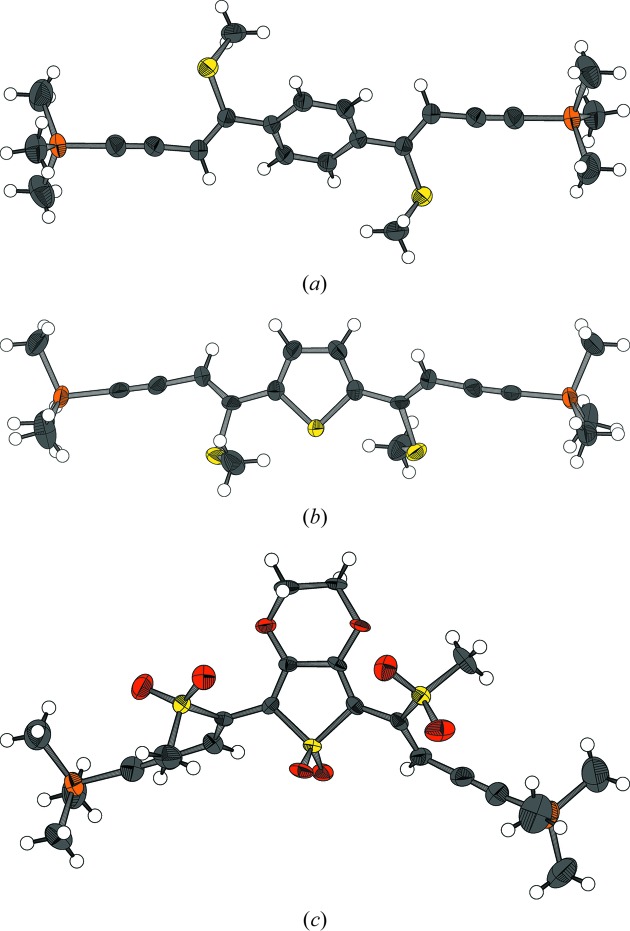
Characteristic geometries of molecules featuring (*a*) a benzene spacer, (*b*) a thiophene spacer and methylthio groups, (*c*) a thiophene dioxide spacer and methylsulfonyl groups. C, O, S and Si atoms are represented by gray, red, yellow and orange ellipsoids drawn at 90% probability levels, H atoms by white spheres of arbitrary radius.

In general, the geometries of the molecules are similar. The most notable trend is that in molecules with a benzene spacer the C=C(—spacer—)C=C torsion angle is 180° (all mol­ecules are symmetric by inversion), *i.e.* the methylthio or methylsulfonyl groups are located at opposite sides of the molecules (Fig. 7 ▸ *a*). In molecules with a thiophene or a thiophene dioxide spacer, on the other hand, the torsion angle is small, *i.e.* the methylthio or methylsulfonyl groups face the same direction. Whereas in the methylthio/thiophene containing molecules [TSEM (**2**), ESEM (**3**) and DSEM (**4**)] the S atoms of the methylthio groups are in close vicinity to the S atom of the thiophene ring (Fig. 7 ▸ *b*), in the oxidized trisulfone compound oxESEM (**3*b***) the methylsulfonyl groups are located at the opposite side of the aromatic ring owing to steric repulsion (Fig. 7 ▸ *c*).

With the exception of the non-spacer-extended molecules [NSEM-TBDMS (**5**), ASYM (**6**)], the methylthio and methylsulfonyl groups feature a distinct inclination to the plane of the ene fragment. In contrast, in NSEM-TBDMS (**5**) and ASYM (**6**) one methylthio unit is nearly perfectly aligned with the ene fragment. Whereas the CH_3_ unit of the methylthio groups is generally turned towards the spacer (C=C—S—CH_3_ torsion angle > 90°), the methylsulfonyl groups face the side chain (C=C—SO_2_—CH_3_ torsion angle < 90°). An exception is polymorph II of oxBSEM (**1*b***), whereby the two unique molecules show the two behaviors, respectively.

The silyl group is in most cases in a *gauche* conformation to the side chain with inclination angles of ∼ 10–30°. Only the TMS groups in one out of two molecules in polymorph II of oxBSEM (**1*b***) and one out of two TMS groups in ASYM (**6**) are in a nearly perfect *anti* position (176.7 and 178.4°, respectively).

### 2.3. Layer stacking   

With the exception of polymorph III of oxBSEM (**1*b***), all structures crystallize in distinct crystallochemical layers, whereby the silyl groups are located at the layer interfaces. In Table 2 ▸ the symmetry of the layers and the operations relating adjacent layers are compiled. Here and in the following text, layer group types are designated with lower case Bravais symbols reflecting the two-dimensionality of the lattice (Kopsky & Litvin, 2006 ▸) and parentheses indicating the direction of missing translation symmetry as is customary in OD theory (Dornberger-Schiff & Grell-Niemann, 1961 ▸).

**Figure 8:**
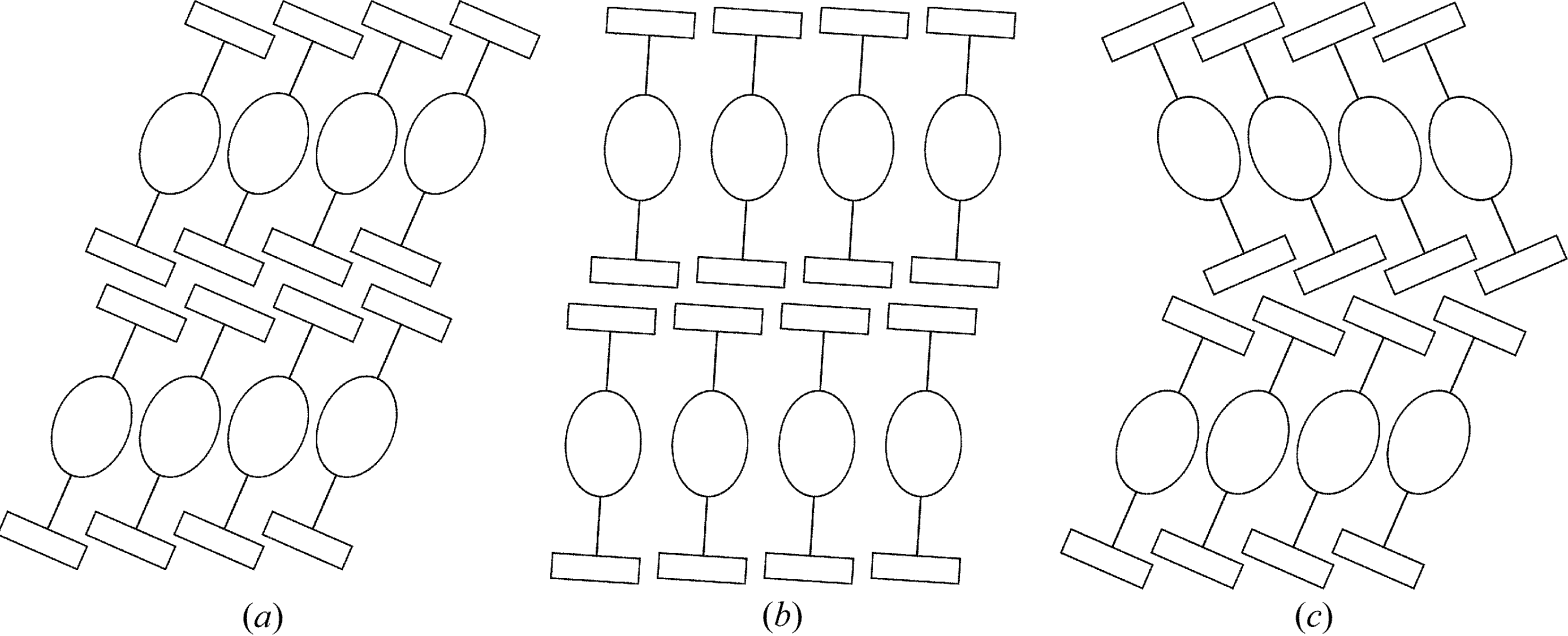
Scheme of the layer contacts of the title compounds observed in (*a*) the common case, (*b*) oxESEM (**3*b***) and (*c*) polytype II of DSEM (**4**) and NSEM-TBDMS (**5**). Silyl groups are represented by rectangles, the yne fragments by lines and the center unit of the molecules (spacer, methylthio/methylsulfonyl and ene fragment) by ellipses.

The main axis of the molecules is in general distinctly inclined with respect to the stacking direction and the molecules in adjacent layers are inclined in the same direction as schematized in Fig. 8 ▸(*a*). In oxESEM (**3*b***), on the other hand, the molecules feature only a little inclination, resulting in a layer stacking comparable to the scheme in Fig. 8 ▸(*b*). The layer stacking in polytype II of DSEM (**4**) and in NSEM-TBDMS (**5**) are exceptions: The molecules in adjacent layers are inclined in opposite directions (Fig. 8 ▸ *c*). Indeed, these two are the only structures presented in this work that lack inversion symmetry relating adjacent layers (Table 2 ▸).

### 2.4. Structural relationships   

**Figure 9:**
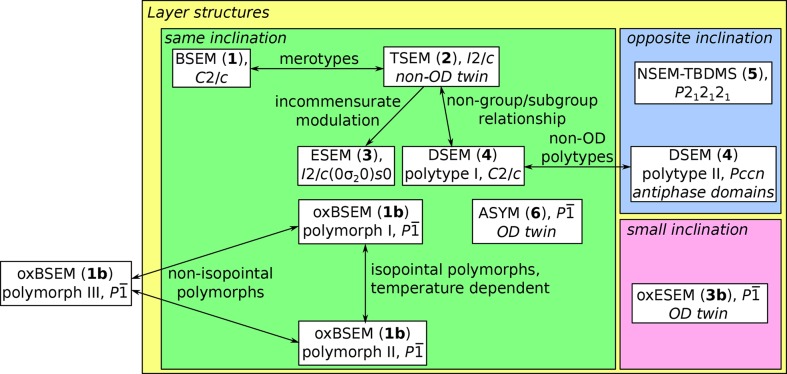
Structural relationships of the crystals under investigation. Structures crystallizing as layers are marked by a yellow backdrop; those with same and opposite inclination in adjacent layers or little inclination (see Fig. 8 ▸) by green, blue and pink backdrops, respectively. Twinning or antiphase domains are indicated in cursive.

Before describing the individual crystal structures in detail (§2.5), here an overview of the structural relationships of the crystals under investigation is given. The relationships and interesting crytallographical features are summarized in Fig. 9 ▸. The 11 structures can be partitioned into two families and three unrelated structures.

The first family is made up of the structures of BSEM (**1**) and of the analogs obtained by substitution of the aromatic spacer [TSEM (**2**), ESEM (**3**) and DSEM (**4**)]. BSEM (**1**) and TSEM (**2**) are merotypes, *i.e.* belong to a family of structures that possess layers common to all members, but also layers found only in certain members (Makovicky, 1997 ▸). Although the term originates from the crystallography of minerals, an interpretation of molecular organic structures in terms of merotypism has for example been given by our group (Stöger, Kautny *et al.*, 2012 ▸).

Increase of the steric bulk of the thiophene spacer in TSEM (**2**) to EDOT in ESEM (**3**) leads to an incommensurately modulated structure with a basic structure isostructural (Kálmán *et al.*, 1993 ▸) with TSEM (**2**). The modulation is a compromise between the need for additional space by the EDOT spacer and the retention of the structure of the interlayer contacts formed by the TMS groups.

On further increase of the steric bulk to dimethoxythiophene in DSEM (**4**), periodicity is restored. Although structurally related, the symmetries of TSEM (**2**) and DSEM (**4**) are not related by a group/subgroup relationship. Crystals of two DSEM (**4**) polytypes were grown in which layers connect in geometrically different ways (non-OD polytypes). Owing to these alternative stacking possibilities, the second polymorph crystallizes as antiphase domains (domains related by translation symmetry; Wondratschek, 1976 ▸), leading to weak diffraction intensities. Different possible kinds of connecting layers are also the likely reason for the non-OD twinning (the interface is geometrically different from the individuals) of TSEM (**2**).

Oxidation of the methylthio groups and removal of the spacer leads to unrelated structures. oxBSEM (**1*b***) exists as three polymorphs making up the second family. Two polymorphs (I and II) are structurally related. Whereas the arrangement of the molecules is retained, one out of two molecules inverts orientation. Thus, although the term is usually reserved for inorganic structures, both polymorphs can be considered isopointal (same space group and Wyckoff positions of molecules; de Faria *et al.*, 1990 ▸), but not isostructural. Polymorph III is structurally unrelated and the only analyzed structure that is not composed of layers.

Of the remaining three structures, oxESEM (**3*b***) and ASYM (**6**) crystallize as OD twins, since their layers possess higher symmetry than adjacent layers. In these twins, the layer interface can be considered as a fragment of a different polytype that is locally equivalent to the twin individuals.

Finally, NSEM-TBDMS (**5**) is the only structure crystallizing in a Sohncke space group (the crystal is enantiomorphic).

### 2.5. Crystal structure details   

#### 2.5.1. BSEM (**1**) and TSEM (**2**)   

**Figure 10:**
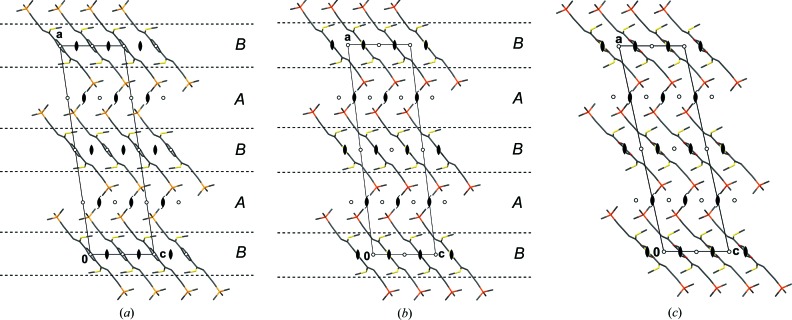
The crystal structures of (*a*) BSEM (**1**), (*b*) TSEM (**2**) and (*c*) the basic structure of ESEM (**3**) viewed down the monoclinic axis [010]. Color codes as in Fig. 7 ▸. H atoms have been omitted for clarity. Symmetry elements with the exception of the glide planes are indicated by the graphical symbols standardized in *International Tables for Crystallography* (Hahn, 2006*a* ▸). Boundaries between the *A* and *B* layers are indicated by dashed lines.

In the structures of BSEM (**1**) and TSEM (**2**) the molecules are arranged in layers parallel to (100) with *p*(1)2/*c*1 symmetry (Figs. 10 ▸ *a* and 10 ▸ *b*). Yet, owing to the intrinsically different symmetries of the *para*-substituted benzene (*mmm*) and 2,5-substituted thiophene (*mm*2) rings, the molecules are located on different Wyckoff positions: Whereas the BSEM (**1**) molecules are symmetric by inversion, the TSEM (**2**) molecules are located on the twofold rotation axes. Adjacent BSEM (**1**) and TSEM (**2**) molecules are related by the mutual operation: twofold rotations for BSEM (**1**) and inversions for TSEM (**2**).

Despite this difference, the outer parts of the layers are virtually identical in both structures. Moreover, adjacent layers connect in equivalent ways *via* 2_1_ screws, *n* glides, inversions and the centering translations (Figs. 10 ▸ *a* and 10 ▸ *b*).

Thus, to relate their symmetry, the crystal structures of BSEM (**1**) and TSEM (**2**), are ‘sliced’ into two kinds of layers, which do not correspond to layers in the chemical sense. The *A* layers [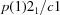], which are composed of the —C C—TMS fragments of adjacent molecules, are equivalent in both structures. The *B* layers [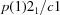] containing the center unit (aromatic rings, ene fragment and methylthio groups) on the other hand are fundamentally different (Figs. 10 ▸ *a* and 10 ▸ *b*).

Since the *A* and *B* layers of both structures crystallize in the same layer group type, BSEM (**1**) and TSEM (**2**) possess the same space-group symmetry. Yet, in a comparable cell setting, the *B* layers are translated along 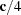 in TSEM (**2**) compared with BSEM (**1**), thus the former is described in the non-standard 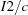 setting of 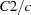.

**Figure 11:**
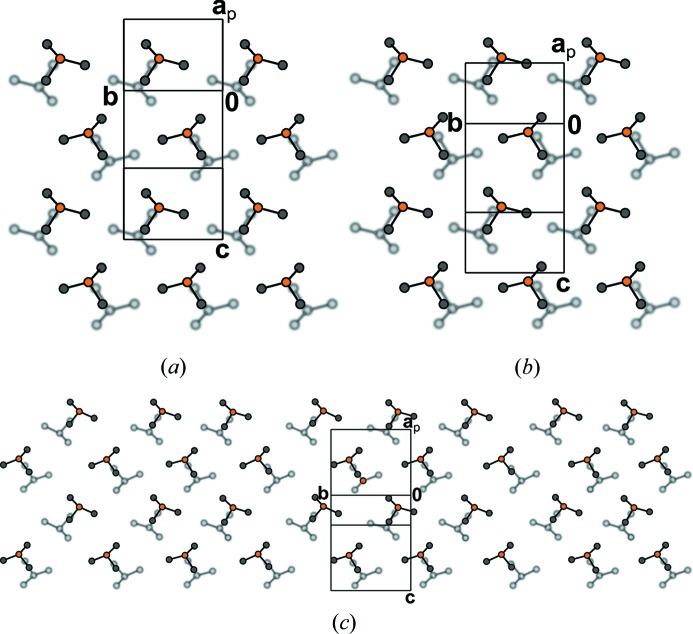
Contact of TMS groups in two adjacent layers of (*a*) BSEM (**1**), (*b*) TSEM (**2**) and (*c*) ESEM (**3**) projected on the layer plane (100). Groups of the lower layer are gray and blurred, other color codes as in Fig. 7 ▸. H atoms have been omitted for clarity. The extent of the unit cell [of the basic structure in the case of ESEM (**3**)] is indicated by black lines.

The BSEM (**1**) molecule is slightly longer than the TSEM (**2**) molecule (Si–Si distance of 16.36 *versus* 16.07 Å). However, since the inclination of the BSEM (**1**) molecules with respect to the layer plane is slightly more pronounced, the molecular layer width is smaller (*a*sinβ/2 = 16.89 *versus* 17.10 Å) and the packing in the [001] direction less dense [*c* = 10.3442 (18) *versus* 10.1978 ▸(8) Å]. The benzene rings require more space in the [010] direction compared with the thiophene rings, as observed by an increased lattice parameter *b* of 6.8690 (12) *versus* 6.7415 (4) Å. These small structural modifications have nearly no impact on the layer interface (Figs. 11 ▸ *a* and 11 ▸ *b*).

The crystal of TSEM (**2**) was twinned by reflection at (001). Often, OD theory is a convenient tool to understand twinning in layered structures (Stöger *et al.*, 2013 ▸): the twin domain is interpreted as an alternative but locally equivalent stacking sequence. Application of OD theory to TSEM (**2**) did not lead to such a convincing interpretation, since no local pseudosymmetry is present. From a crystallochemical point of view, the only plausible twin interface is the boundary of the molecular layers. The molecule contact would then resemble more closely Fig. 8 ▸(*c*) than Fig. 8 ▸(*a*). Thus, the twin interface is geometrically different from the twin individuals. The possibility of such a twinning is demonstrated by the DSEM (**4**) polytypes (§2.5.3).

#### 2.5.2. ESEM (**3**)   

The basic structure of ESEM (**3**) is isostructual with TSEM (**2**) (Fig. 10 ▸ *c*). Compared with TSEM (**2**), the ESEM (**3**) molecules are more strongly inclined with respect to the layer plane, resulting in a larger monoclinic angle of β = 102.301 (2)° *versus* β = 96.889 (5)° and smaller layer widths (*a*sinβ = 31.72 *versus* 34.19 Å). As expected, the lattice parameter *b* increases significantly from 6.7415 (4) to 8.4003 (5) Å owing to the additional space needed by the ethyl­ene­di­oxy group.

The actual structure is incommensurately modulated with a modulation wavevector of **q** = σ_2_ b* with σ_2_ = 0.6223 (1) ≃ 5/8. Although incommensurately modulated structures are non-periodic, they can be conveniently described by embedding into 3+*n* superspace (Janssen *et al.*, 2007 ▸; van Smaalen, 2007 ▸). The superspace of ESEM (**3**) has 3+1-dimensional superspace group symmetry (van Smaalen *et al.*, 2013 ▸; Stokes *et al.*, 2011 ▸) 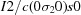, a non-standard setting of 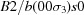, No. 15.3 (Janssen *et al.*, 2006 ▸).

Since **q** is parallel to the layer planes, the layers are equivalent. Adjacent layers are related by a 2_1_ screw with intrinsic translation along **a**_s2_/2 + **a**_s4_/2, corresponding to an increase of the internal coordinate *t* by (σ_2_ + 1)/2.

The twofold rotation of the molecules in the basic structure features an intrinsic translation along **a**_s4_/2 in internal space. Thus, half of each molecule is completed by a second half located at *t* + 1/2. The individual molecules are therefore generally not symmetric by twofold rotation and the actual S1 atoms are not located on the twofold rotation axis.

**Figure 12:**
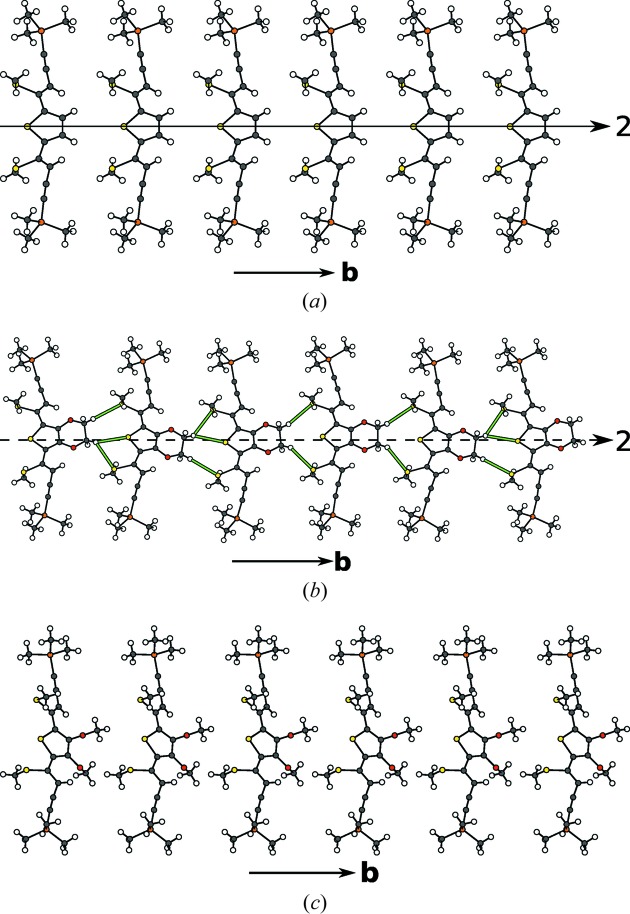
Chain of (*a*) TSEM (**3**), (*b*) ESEM (**3**) and (*c*) DSEM (**4**) molecules running along [010]. Color codes as in Fig. 7 ▸. In (*b*) intermolecular H⋯S contacts up to 2.91 Å are indicated by green rods to highlight the different types of intermolecular contacts. An arrow indicates a twofold rotation of the chain. If it is dashed it is valid only for the basic structure.

In Figs. 12 ▸(*b*) and 12 ▸(*c*) the progression from the unmodulated chains of molecules along [010] in TSEM (**2**) to the modulated chains in ESEM (**3**) is depicted. On the one hand, the steric repulsion of the ethyl­ene­dioxy groups and the S atoms requires more space in the [010] direction, on the other hand the layer contacts *via* the TMS groups remain similar to those of TSEM (**2**) (Figs. 11 ▸ *b* and 11 ▸ *c*). To accommodate for both, the structure reacts by different rotations of adjacent molecules in an incommensurate way.

**Figure 13:**
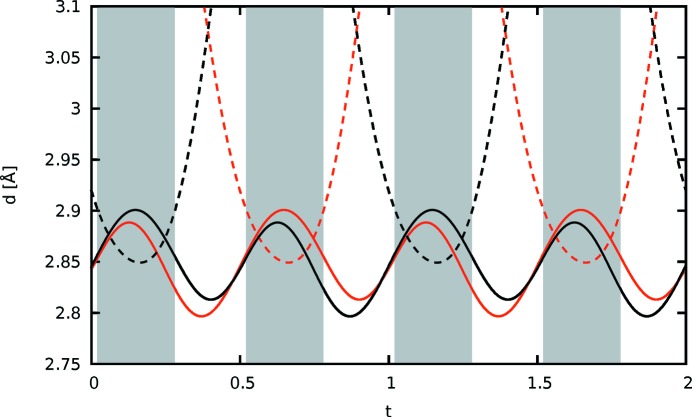
Distance of the equatorial H112 atoms of the ethylenedioxy bridge in ESEM (**3**) to the S atoms in the adjacent molecules plotted against *t*. The curves of the two H112 atoms are red and black, respectively. The distances to the S atoms of the methylthio groups and the thiophene ring are drawn using continuous and dashed lines, respectively. Gray backdrops mark the ranges where an H atom protrudes into the cavity formed by the three S atoms of the adjacent molecule.

The distance of the equatorial H112 atoms of the ethyl­ene­dioxy group to the S atoms is plotted against the internal coordinate *t* in Fig. 13 ▸. Roughly two regions can be distinguished. For approximately half of the *t* values, marked by a gray backdrop in Fig. 13 ▸, an H112 atom is close to the thiophene S. Adjacent molecules are inclined to each other and the H112 atom protrudes into the cavity defined by the three S atoms. For the remaining *t* values, the molecules feature little inclination and the two H112 atoms connect only to the S atoms of the methylthio atom. In Fig. 12 ▸(*b*) H—S distances up to an arbitrary value of 2.91 Å are indicated to highlight the two kinds of contacts.

**Figure 14:**
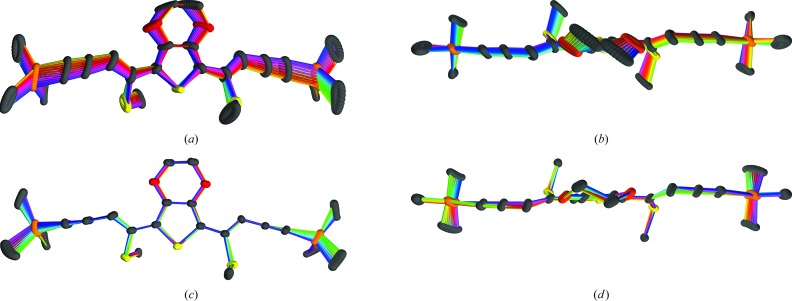
Overlap of an ESEM (**3**) molecule at 24 equidistant *t* values (*a*, *c*) projected approximately on the molecular plane and (*b*, *d*) viewed down the twofold axis of the basic structure. In (*a*, *b*) the orientation of the molecules from the actual structure is unchanged; In (*c*, *d*) the molecules are rotated to minimize the interatomic distances of the thiophene rings. Bond colors with similar hue signify close *t* values, complementary colors a shift of *t* + 1/2.

Owing to the rigidity of the ESEM (**3**) molecules small rotations of the EDOT core translate into larger displacements of the TMS groups (Figs. 14 ▸ *a* and 14 ▸ *b*). Therefore, the connection of adjacent layers *via* the TMS groups features a wide variation of interatomic distances (Fig. 11 ▸). This surprising flexibility of the interlayer contacts enables incommensurate modulation.

**Figure 15:**
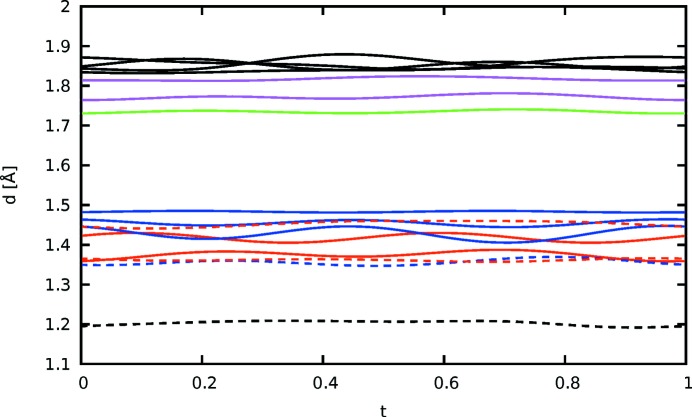
All intramolecular bond lengths in ESEM (**3**) involving non-H atoms plotted against *t*. Symmetry-equivalent distances located at *t* + 1/2 are not listed. Color codes: Si—C: black; C—S aliphatic: pink; C—S aromatic: green; C—C single bond (spacer to ene, ene to yne and yne to TMS): blue; C—O: red dashed; C—C aromatic: red; C—C double bond: blue dashed; C—C triple bond: black dashed.

The variation of the geometry of the ESEM (**3**) molecules is pictured in Figs. 14 ▸(*c*) and 14 ▸(*d*). The interatomic distances are close to constant (Fig. 15 ▸) and in good agreement with the expected values (Allen *et al.*, 2006 ▸). Whereas the core of the molecule is virtually identical in all molecules, the side arms (yne fragment, TMS group) feature significant bending (Fig. 14 ▸ *c*), needed to contact adjacent layers.

#### 2.5.3. DSEM (**4**)   

Like in TSEM (**2**) and ESEM (**3**), the DSEM (**4**) molecules in both polytypes are arranged in rods running along the [010] direction (Fig. 12 ▸ *c*). In contrast to ESEM (**3**), periodicity in the [010] direction is restored by rotating all molecules in a rod in the same direction (Fig. 12 ▸ *c*). The symmetry of the rods is thus reduced from 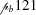 to 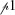. The rods form pairs which are related by inversion and adjacent pairs are related by 2_1_ screws. As a consequence the **c** lattice vector is doubled compared with TSEM (**2**). Owing to the different arrangements of the rods the DSEM (**4**) layers cannot be considered as superstructures of the TSEM (**2**) layers and indeed, their symmetry groups (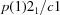 with doubled **c** and 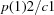) are not related by a group/subgroup relationship.

Although the DSEM (**4**) polytypes are non-OD polytypes, in the following discussion the naming conventions of OD theory will be used (Ferraris *et al.*, 2008 ▸): the layers are designated as *A_n_*, whereby the *n* is a serial index. **a**_0_ is the vector normal to the layer planes with the length of one layer thickness.

**Figure 16:**
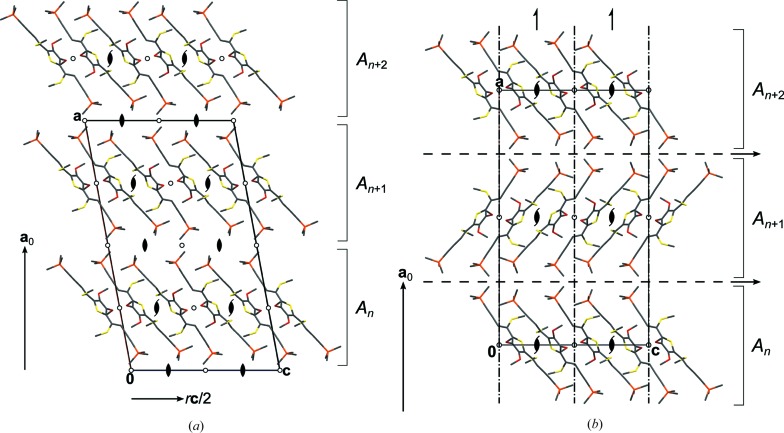
The crystal structures of polytypes (*a*) I and (*b*) II of DSEM (**4**) viewed down [010]. Color codes and symbols as in Fig. 7 ▸. H atoms have been omitted for clarity. The *A_n_* layers are indicated to the right by brackets.

Given an *A_n_* layer, the adjacent *A*_*n*+1_ layer can appear in two different orientations. *A_n_* and *A*_*n*+1_ are either related by the operations listed in Table 2 ▸, line 4, or those in line 5. The symmetry elements are indicated in Fig. 16 ▸.

**Figure 17:**
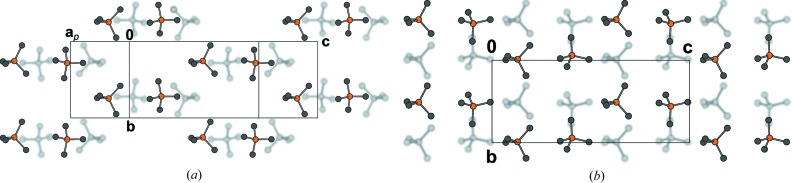
Geometrically non-equivalent contacts of two layers in polytypes (*a*) I and (*b*) II of DSEM (**4**) projected on the layer plane (100). The TMS groups and the connecting *sp* ^1^ hybridized C atom are shown. Atoms of the lower layer are gray and blurred, color codes of the top layer as in Fig. 7 ▸. H atoms have been omitted for clarity.

Thus, the layers can be connected to an infinity of polytypes, which are not OD polytypes because (*A_n_*, *A*_*n*+1_) layer pairs are not necessarily equivalent [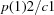 and 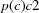 symmetry, respectively]. The polytypes differ from other non-OD polytypes we discussed before (Stöger *et al.*, 2012*a* ▸; Stöger & Weil, 2013 ▸). In the latter, which we designated as ‘non-classic OD’ polytypes, every polytype is at every point locally equivalent to all other polytypes, *i.e.* every point belongs to at least two equivalence regions (Grell, 1984 ▸). In DSEM (**4**), on the other hand, the contact plane of the layers differs geometrically among polytypes as depicted in Fig. 17 ▸. As in the case of ESEM (**3**) this demonstrates a remarkable flexibility of the layer contacts and confirms the assumption that the twinning of TSEM (**2**) is likewise caused by non-equivalent layer contacts.

Although the symmetry groupoids of these kind of non-OD polytypes were not elaborated up to now, the OD concept of polytypes with a *maximum degree of order* (MDO) (Dornberger-Schiff, 1982 ▸) can nevertheless be applied. There are two polytypes that cannot be decomposed into simpler polytypes. They are generated by continuous application of either set of operations relating the adjacent layers. The MDO_1_ polytype has *C*2/*c* symmetry and lattice vector 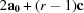; MDO_2_ *Pccn* symmetry and lattice vector 2**a**_0_.

The observed polytypes I and II are MDO_1_ and MDO_2_, respectively. Indeed, it is well documented for OD structures that ordered polytypes are in the vast majority of cases MDO. Fragments of the MDO_2_ polytype in MDO_1_ result in twinning by reflection at a plane normal to [001]. No such twinning was observed in the investigated crystal. Stacking faults in MDO_2_, on the other hand, results in antiphase domains (Wondratschek, 1976 ▸), since the MDO_2_ domains are related by translation. Although in principle not directly observable in diffraction patterns, we suspect that such stacking faults exist and cause the systematic low scattering power of the polytype II crystals.

**Figure 18:**
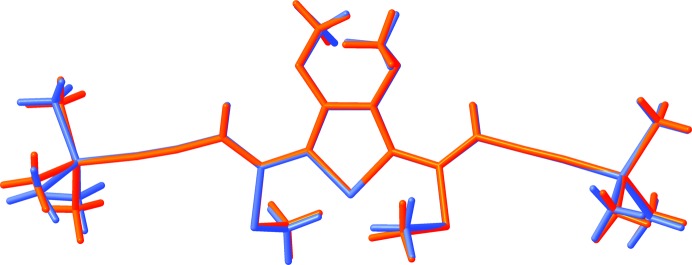
Overlap of the DSEM (**4**) molecules in polytypes I and II, drawn in red and blue, respectively.

As opposed to OD structures, where ordered polytypes usually feature desymmetrization of the layers compared to the idealized description (Ďurovič, 1979 ▸), the *A* layers in both MDO polytypes of DSEM (**4**) possess the 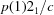 symmetry of the idealized description. A deviation from the idealized model is nevertheless observed by a slight variation of the lattice parameters and layer widths across structures [*a*sinβ = 33.765 *versus* 33.630 (10) Å, *b* = 8.1665 (5) *versus* 8.271 (2) Å and *c* = 20.0791 (12) *versus* 19.717 (6) Å] and a small deviation of the molecular conformations (Fig. 18 ▸). As expected, the largest deviation is observed for the TMS groups, which are located in geometrically different environments in both polytypes.

#### 2.5.4. oxBSEM (**1*b***), polymorphs I and II   

**Figure 19:**
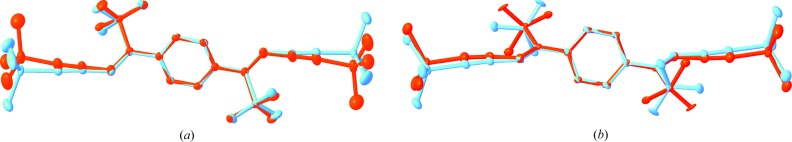
Overlap of the independent oxBSEM (**1*b***) molecules in (*a*) the high-temperature polymorph I and (*b*) the low-temperature polymorph II. The molecules are drawn in red and blue, respectively. H atoms have been omitted for clarity.

Both polymorphs consist of two crystallographically different oxBSEM (**1*b***) molecules (Fig. 19 ▸), called *A* and *B*, both located on centers of inversion (*Z*′ = 2/2). In both polymorphs, the crystallographically independent molecules feature different conformations: The molecules in polymorph I differ by the conformation of the TMS groups with respect to the remaining molecule, whereas in polymorph II the major difference pertains to the orientation of the methylsulfonyl groups (Fig. 19 ▸). Nevertheless, the torsion angle differences between all four conformations (Table 1 ▸) are too small for the molecules to be considered as conformers according to the criteria of Cruz-Cabeza & Bernstein (2014 ▸). Thus, the changes in these polymorphs are only conformational adjustments, though to a rather large degree in the molecule of polymorph II that has a different orientation of the methylsulfonyl groups.

**Figure 20:**
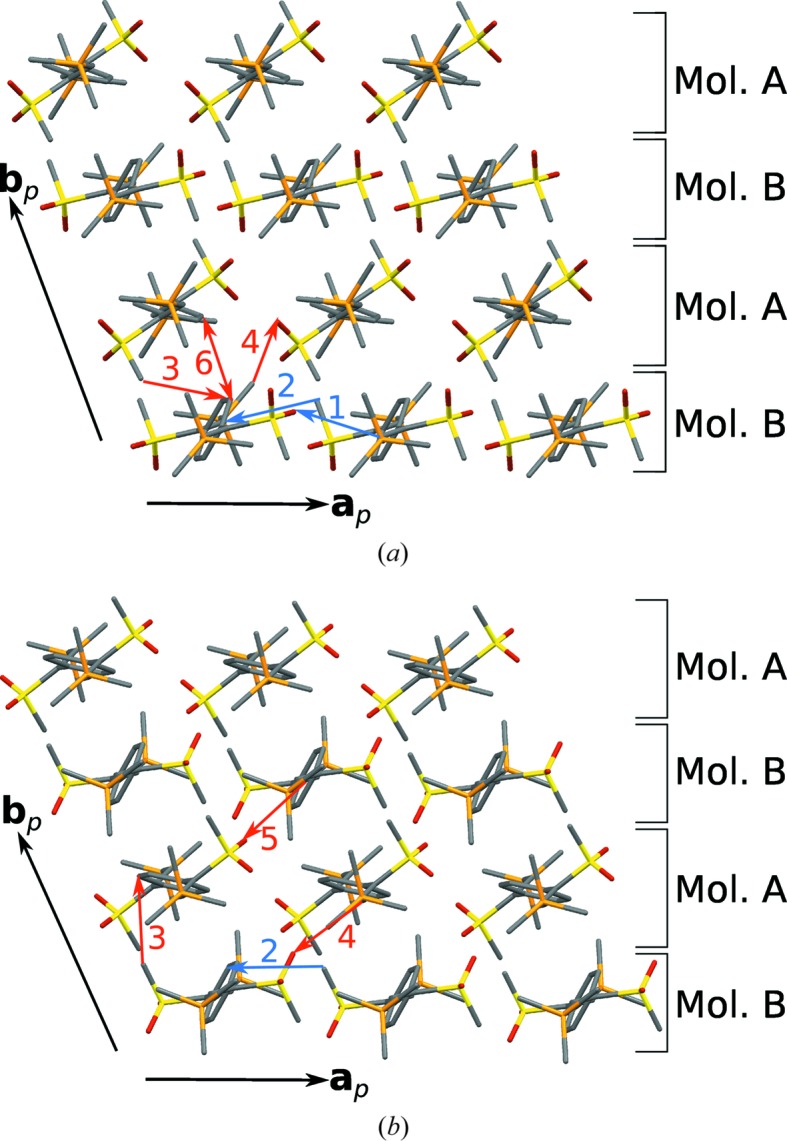
Layers in polymorphs (*a*) I and (*b*) II of oxBSEM (**1*b***) viewed approximately along the main axis of the molecules. Color codes as in Fig. 7 ▸. H atoms have been omitted for clarity. C—H⋯O and C—H⋯C contacts are indicated by arrows originating from the ‘donor’ C atoms, C—H⋯H—C contacts by double-sided arrows connecting the C atoms.

The molecules are arranged in layers parallel to (001) with 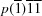 symmetry, whereby the *B* molecules form rods along [100], connected by the *A* molecules (Fig. 20 ▸).

**Figure 21:**
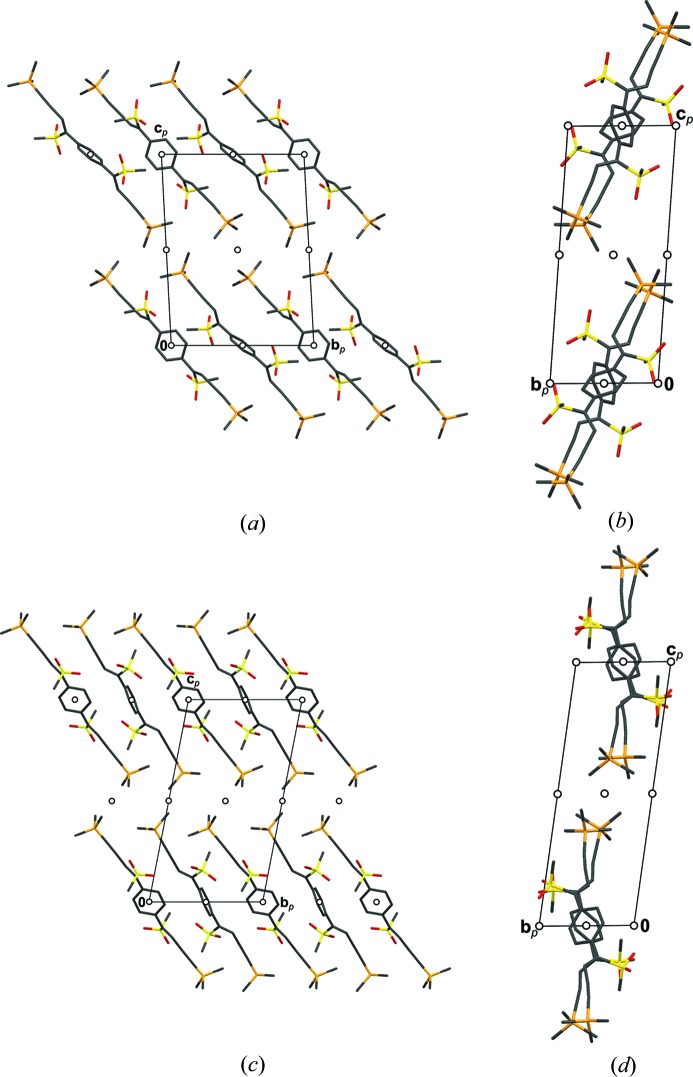
Crystal structures of the polymorphs (*a*, *b*) I and (*c*, *d*) II of oxBSEM (**1*b***), viewed down (*a*, *c*) [010] and (*b*, *d*) [100]. Color codes as in Fig. 7 ▸. H atoms have been omitted for clarity.

The most striking difference between the two polymorphs is the orientation of the *A* molecules, which are rotated by nearly 180°. In projection along [100], the S atoms of the methylsulfonyl groups are nearly superimposed in polymorph II, while in polymorph I the methylsulfonyl groups of subsequent molecules point in opposite directions (Fig. 21 ▸).

**Figure 22:**
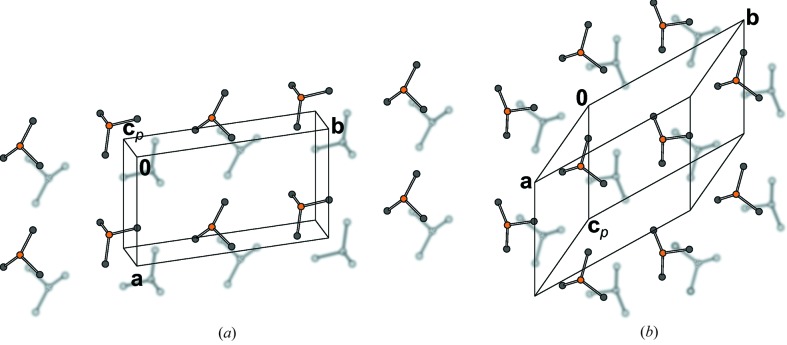
Layer contacts in the polymorphs (*a*) I and (*b*) of oxBSEM (**1*b***) projected on the layer plane (100). Only the TMS groups are shown. Atoms of the lower layer are gray and blurred, color codes of the top layer as in Fig. 7 ▸. H atoms have been omitted for clarity.

Thus, the I 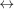 II phase transition has to be considered reconstructive, which is consistent with the destruction of large single crystals on cooling. The transformation is accompanied by an inclination of the molecules with respect to the stacking direction (Figs. 21 ▸ *b* and 21 ▸ *d*). In consequence, the layer interfaces are fundamentally different in the two polytypes (Fig. 22 ▸), demonstrating again the flexibility in layer arrangements allowed by the TMS groups.

**Figure 23:**
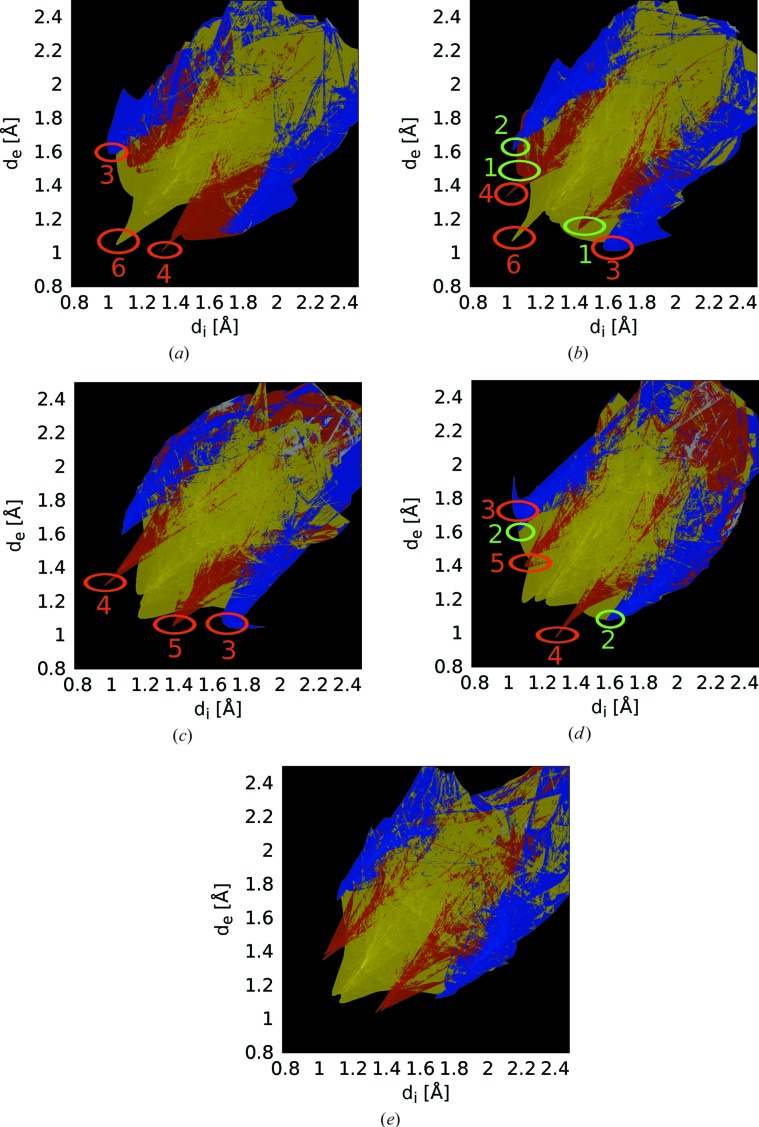
*d*_i_/*d*_e_ fingerprint plots of the oxBSEM (**1*b***) molecules in polymorphs (*a*, *b*) I, (*c*, *d*) II and (*e*) III, calculated with *CrystalExplorer* (Wolff *et al.*, 2012 ▸). Regions where H⋯H, H⋯O and H⋯C dominate are drawn in yellow, red and blue, respectively, other regions in gray. Brighter colors indicate a higher proportion of the surface. Regions discussed in the text connecting two *B* molecules or *A* and *B* molecules are marked by green and red ellipses, respectively.

Although methylsulfonyl groups are potential hydrogen bond acceptors, the oxBSEM (**1*b***) molecules do not possess classical hydrogen-bond donors. Indeed, attempts to analyze the phase transition by listing the weak hydrogen bonds of the two polymorphs were inconclusive, since these lists depend on rather arbitrary distance and angle limits. A more holistic and unbiased approach for the description of molecular inter­actions in polymorphs, which was established in the last decade, is the analysis of molecular Hirshfeld surfaces (Spackman & Jayatilaka, 2009 ▸). The *d*_e_/*d*_i_ fingerprint plots (Spackman & McKinnon, 2002 ▸) of the two molecules in both polymorphs are depicted in Fig. 23 ▸. As expected, contacts not involving H atoms, as well as those involving S and Si atoms, are negligible. First conclusions can be drawn from the shape of the plots: In both polymorphs, the individual plots are not symmetric by reflection at the *d*_e_ = *d*_i_ line, but the plots of the crystallographically independent molecules are approximately related by such an operation. Thus, the closest contacts are mostly between non-equivalent molecules along the [010] direction. An exception are the regions 1 and 2 in Fig. 23 ▸, which correspond to C=C—H⋯O and SC—H⋯C C contacts of equivalent molecules along [100] (Fig. 20 ▸).

Surprisingly, the fingerprint plot of the *A* molecule of polymorph I resembles the plot of the *B* molecule of polymorph II and *vice versa*. Thus, one could say that the roles of the donor and acceptor are reversed on phase transition, although overall the type of interatomic interaction remains similar. The most prominent interactions are indicated in Fig. 23 ▸ and correlated to the actual atoms in Fig. 20 ▸ and Table 3 ▸. A striking feature that is only observed in polymorph I is region 6, a very short C—H⋯H—C contact (2.30 Å) between two aromatic protons. Thus, although there is no definite proof, one might speculate that, on cooling, the structure contracts until these H atoms are too close and the structure becomes unstable. This conjecture would not have been insinuated without the analysis of fingerprint plots.

#### 2.5.5. oxBSEM (**1*b***), polymorph III   

**Figure 24:**
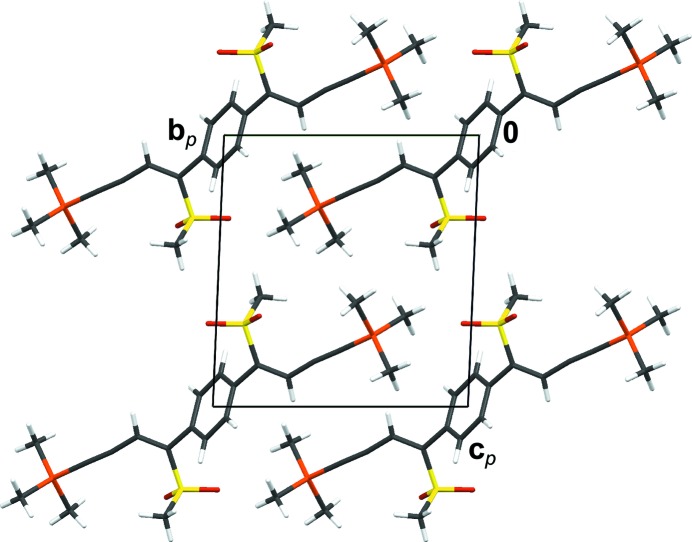
Crystal structure of polymorph III of oxBSEM (**1*b***) viewed down [100]. Color codes as in Fig. 7 ▸.

The molecules in polymorph III of oxBSEM (**1*b***) are not arranged in distinct silyl-group delimited layers (Fig. 24 ▸). One crystallographically unique oxBSEM (**1*b***) molecule is located on a center of inversion. It can again not be considered a different conformer. As expected, the Hirshfeld fingerprint plot (Fig. 21 ▸ *e*) is nearly symmetric by reflection at *d*_e_ = *d*_i_. It most closely resembles the plot of the *A* molecule in polymorph II, but the H⋯C contacts are distinctly less prominent, indicating an energetically more favorable packing. Indeed, polymorph III has higher symmetry (same space group type, but *Z*′ = 1/2 *versus* 2/2) and distinctly higher density (1.267 *versus* 1.229 g cm^−3^ at 100 K). Thus, the I 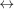 II transition is an example of Ostwald’s rule stating that a system does not change into the thermodynamically stable, but the nearest metastable state.

#### 2.5.6. oxESEM (**3*b***)   

**Figure 25:**
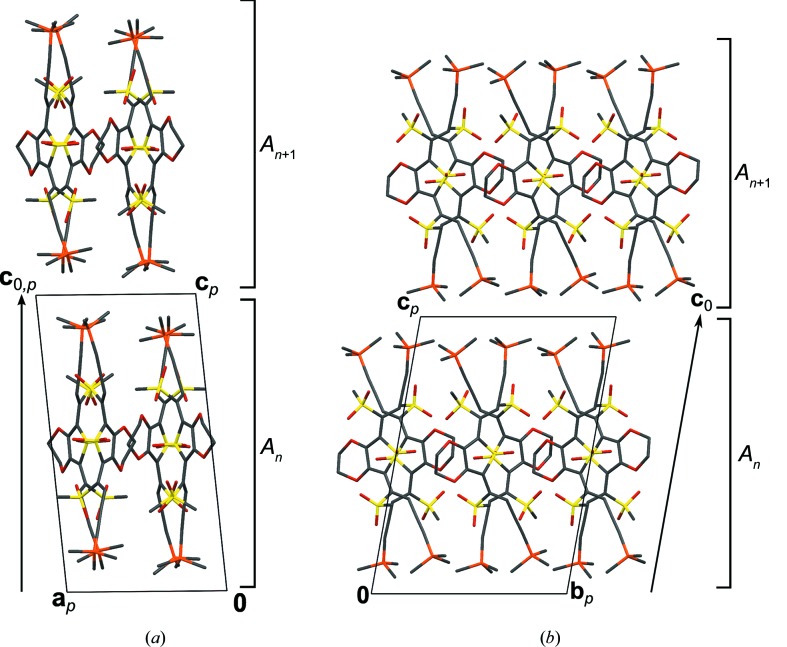
The crystal structure of oxESEM (**3*b***) viewed down (*a*) [010] and (*b*) [100]. The location of the *A_n_* layers is indicated by brackets to the right. Color codes as in Fig. 7 ▸.

The oxESEM (**3*b***) molecules are arranged in layers parallel to (001) with (idealized) 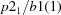 symmetry (Fig. 25 ▸), which are, despite possessing the same layer group type, structurally unrelated to the layers in BSEM (**1**), TSEM (**2**), the basic structure of ESEM (**3**) and DSEM (**4**).

One crystallographically unique molecule is located on a general position. The layers are stacked in such a way that the *b*-glide planes do not overlap. In consequence oxESEM (**3*b***) belongs to a category I OD family composed of layers of one kind. The OD groupoid family symbol reads according to the notation introduced by Dornberger-Schiff & Grell-Niemann (1961 ▸) as
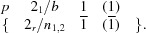
It has to be noted that in this case **c**_0_ is conveniently chosen not normal to the layer planes, to reflect the monoclinic point group 2/*m*11 of the OD family (Fichtner, 1979 ▸), whereby a second metric parameter describing the relative layer positions vanishes. In one possible arrangement of the (*A_n_*, *A*_*n*+1_) layer pair, *A*_*n*+1_ is related to *A_n_* by a 2_*r*_ screw with intrinsic translation along *r* **a**/2 or equivalently by an *n*_1,2_ glide with intrinsic translation along (**b**/2) + **c**_0_. The other geometrically equivalent arrangements are derived using the *NFZ* relationship (Ďurovič, 1997 ▸): Given an *A_n_* layer, an adjacent 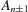 layer can appear in *Z* = *N*/*F* = [*p*11(1):*pb*1(1)] = 2 orientations, related by the *b* glides of *A_n_*. *p*11(1) and *pb*1(1) are the groups of those layer operations that do not invert the orientation of the layers with respect to the stacking direction [called λ-τ partial operations (POs) in the OD literature].

**Figure 26:**
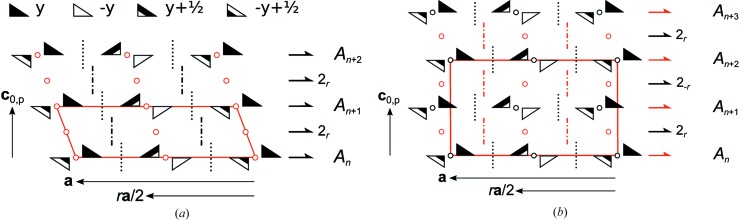
Schematic representation of the symmetry of the (*a*) MDO_1_ and (*b*) MDO_2_ polytypes of oxESEM (**3*b***). Triangles are black on one and white on the other side. (Partial) symmetry operations of a layer and relating adjacent layers are indicated by the graphical symbols standardized in *International Tables for Crystallography* (Hahn, 2006*a* ▸). Additionally for operations with non-crystallographic intrinsic translations the printed symbol is given.

These stacking possibilities give rise to two MDO polytypes: MDO_1_ {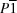, **c** = **c**_0_ + [(*r* − 1)/2]**a**} and MDO_2_ (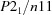, **c** = 2**c**_0_), obtained by continuous application of the 2_*r*_ screws and *n*_1,2_ glides, respectively. The symmetry of the two polytypes is schematized in Fig. 26 ▸.

The major polytype of the crystals under investigation is the MDO_1_ polytype. Fragments of the MDO_2_ polytype were observed indirectly by systematic twinning. The twin element corresponds to the plane of the *b* glides of the *A* layers. This kind of twinning is fundamentally different from that in TSEM (**2**) or the polytypism of DSEM (**4**). The latter is only possible owing to the flexibility of the layer contacts, whereas in oxESEM (**3*b***) the layer contacts are equivalent in all polytypes.

In the major MDO_1_ polytype, the symmetry of the *A* layers is reduced by an index of 2 from 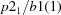 to 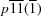. This translates to a small deviation of γ = 89.771 (2)° from the ideal value of 90° imposed by the rectangular layer lattice and a small deviation of the atoms from the positions compared with the idealized 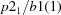 layers. Significant deviations from ideal symmetry are limited to the TMS groups, which are located at the layer interfaces [deviations of 0.272 Å (Si2) up to 0.508 Å (C10)]. This is expected, since the layer interfaces are located in an environment which deviates from the ideal layer symmetry. The closer the atoms are located to the center of the layers, the smaller the deviation. The C atoms of the yne fragment connecting to the TMS group deviate by 0.130 Å (C8) and 0.138 Å (C16), all other atoms by less than 0.100 Å.

#### 2.5.7. NSEM-TBDMS (**5**)   

Although achiral, the NSEM-TBDMS (**5**) mol­ecules crystallize in the Sohncke group 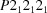. The crystal under investigation was enantiomerically pure [Flack parameter 0.03 (3)]. An estimation of the number of achiral molecules crystallizing in Sohncke groups was given by Pidcock (2005 ▸).

**Figure 27:**
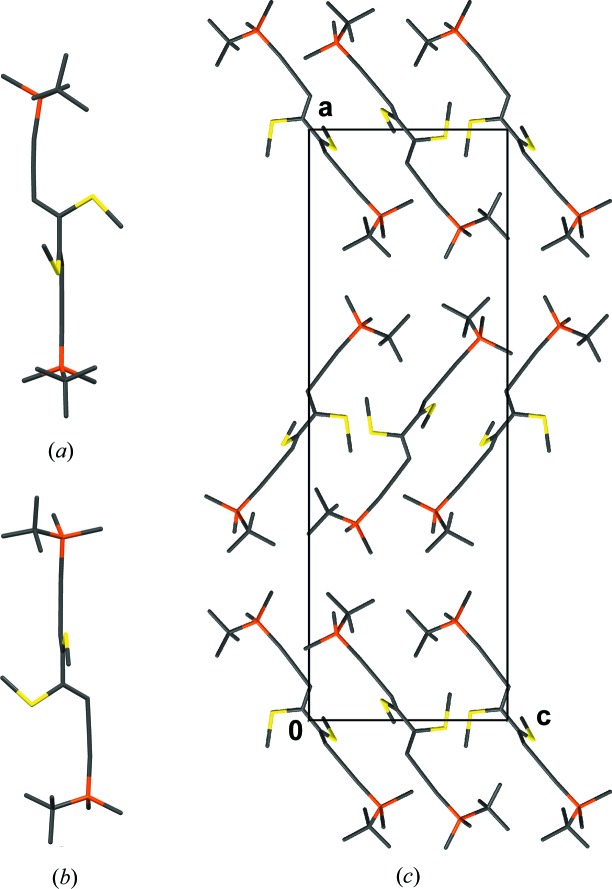
(*a*, *b*) The NSEM-TBDMS (**5**) molecule viewed down two different directions, showing the different conformations of the methylthio group with respect to the TBDMS groups (top group: *gauche*, bottom group: *eclipsed*) and (*c*) crystal structure of NSEM-TBDMS (**5**) viewed down [100]. H atoms have been omitted for clarity. Color codes as in Fig. 7 ▸.

Whereas the central part of the molecule is nearly symmetric by twofold rotation, the TBDMS groups feature a distinctly different orientation with respect to the methylthio groups, resulting in molecules with 1 symmetry (Figs. 27 ▸ *a* and 27 ▸ *b*). The molecules are arranged in layers parallel to (001) with 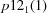 symmetry. The layers in turn are connected by 2_1_ screws with axes parallel to [100] and [010] (Fig. 27 ▸ *c*).

#### 2.5.8. ASYM (**6**)   

**Figure 28:**
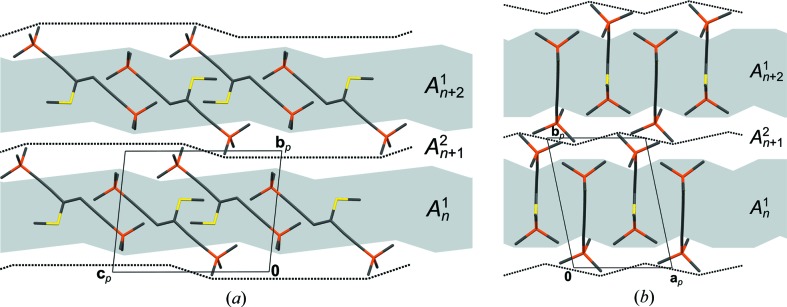
The crystal structure of ASYM (**6**) viewed down (*a*) [100] and (*b*) [001]. The *A* ^1^ and *A* ^2^ OD layers are marked by a gray and white backdrop, respectively. The boundaries of the crystallochemical layers are indicated by dotted lines. Color codes as in Fig. 7 ▸.

The ASYM (**6**) molecules are located on general positions. Despite being polar with respect to the main direction, they are arranged in nonpolar layers parallel to (010) with 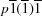 symmetry (Fig. 28 ▸).

In contrast to the other layered structures, the TMS groups are not as clearly located at the layer interface: every second group is moved away from the surface into the layers. This can be attributed to ASYM (**6**) being the shortest of the molecules under investigation.

The systematic twinning of ASYM (**6**) can be explained by local pseudosymmetry: With the exception of one TMS group, the molecules are practically symmetric by reflection at (100) (Fig. 28 ▸ *b*). Thus the structure can be ‘sliced’ into OD layers (Grell, 1984 ▸) of two kinds, which do not correspond to layers in the crystallochemical sense (Fig. 28 ▸). The *A* ^1^ layers contain the parts of the molecule that possess mirror symmetry, whereas the *A* ^2^ layers are made up of the remaining TMS groups.

As a consequence, the structure belongs to a category IV OD family composed of nonpolar layers of two kinds. The corresponding OD groupoid family symbol reads according to the notation introduced by Grell & Dornberger-Schiff (1982 ▸) as
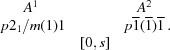
Accordingly, the structure is made up of an alternating stacking of *A* ^1^ and *A* ^2^ layers, with 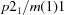 and 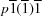 symmetry, respectively. **b**_0_ is chosen not normal to the layer planes so that one metric parameter vanishes. In one possible arrangement of the 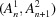 layer pair, the origin of *A* ^2^ is reached from the origin of *A* ^1^ by translation along (**b**_0_/2) + *s* **a**.

According to the *NFZ* relationship, given an 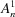 layer, the adjacent 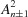 layers can appear in *Z* = *N*/*F* = [*pm*(1)1:*p*1(1)1] = 2 orientations, related by the *m* operation of the 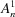 layer. For the *A* ^2^ layers on the other hand, there is only one way to connect to the *A* ^1^ layers, since all λ-τ POs of *A* ^2^ (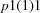) apply likewise to *A* ^1^.

**Figure 29:**
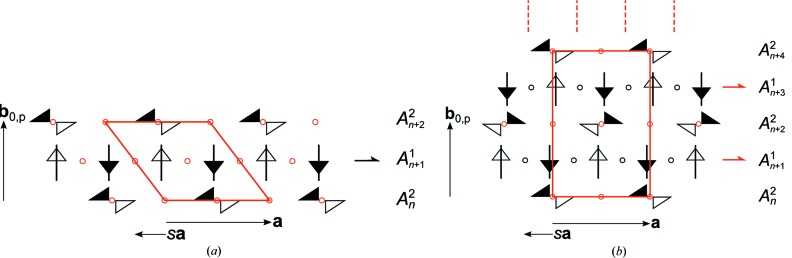
Schematic representation of the symmetry of the (*a*) MDO_1_ and (*b*) MDO_2_ polytypes of ASYM (**6**). Symbols as in Fig. 26 ▸.

These stacking possibilities give rise to two MDO polytypes: MDO_1_ (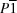, **b** = **b**_0_ + 2*s* **a**) is obtained by continuous application of the inversion operations of the *A* ^1^ layers; MDO_2_ (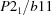, **b** = 2**b**_0_) by application of the 2_1_ screws. The symmetry of the two polytypes is schematized in Fig. 29 ▸.

The bulk of the ASYM (**6**) crystals under investigation are made up of the MDO_1_ polytype, whereas fragments of the MDO_2_ polytype are located at the twin interface. A twin element corresponds to the mirror plane of the *A* ^1^ layers. Again, all polytypes are locally equivalent and no flexibility of the layer contact is needed for twinning.

In MDO_1_, the symmetry of the *A* ^1^ layers is reduced by an index of 2 from 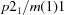 to 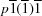. This is reflected by a deviation of β = 92.510 (2)° from the ideal value of 90° according to the rectangular layer lattice, and by a slight deviation of the molecules from their idealized positions. Under the assumption of β = 90°, the only non-negligible deviations (> 0.1 Å) of non-H atoms from the idealized positions of the *A* ^1^ layers are observed for the atoms of the TMS group that are not located on the mirror plane (C10, C11, deviation of 0.11 Å) and for the C3 atom, which connects to the TMS group in the *A* ^2^ layer (deviation of 0.12 Å).

The deviation of β from the ideal value of 90° by 2.51° is remarkably large and distinctly larger than in the case of oxESEM (**3*b***). As a consequence, the lattices of the twin domains do not match (deviation of 5°) and the crystals are distinctly distorted at the twin interface. The orthorhombic MDO_2_ fragment at the twin interface possesses an ideal angle of 90° and it therefore enables the passage of the two extremes of the MDO_1_ domains.

In contrast to oxESEM (**3*b***), the desymmetrization does not result in two crystallographically unique molecules, but rather in a desymmetrization of the *A* ^1^ parts of the molecule from *m* to 1 symmetry.

## 3. Experimental   

Detailed syntheses and spectroscopic characterizations of all compounds are given by Lumpi (2013 ▸). Single crystals of BSEM (**1**) (*i*-PrOH, EtOH), oxBSEM (**1*b***) (EtOH), ESEM (**3**) (EtOH, in a glove-box with N_2_ atmosphere), oxESEM (**3*b***) (MeOH) and DSEM (**4**) (EtOH) were obtained by slow evaporation at room temperature. Crystallization of TSEM (**2**) from solvents failed to give single crystals suitable for single-crystal diffraction. Tiny single crystals were instead afforded by crystallization of the oily sample at ∼293 K over a time period of several months.

Single-crystal data were collected and processed on a Bruker Kappa APEXII diffractometer system (Bruker, 2008 ▸). Data were reduced using the *SAINT-Plus* (Bruker, 2008 ▸) and *EVAL* (Duisenberg *et al.*, 2003 ▸) suites and corrected for absorption effects with *SADABS* or *TWINABS* (Bruker, 2008 ▸). Structures were solved with *SUPERFLIP* (Palatinus & Chapuis, 2007 ▸) and refined with *JANA*2006 ▸ (Petříček *et al.*, 2014 ▸). More details on single-crystal diffraction and structure refinement are available as supplementary materials.

## 4. Conclusion and outlook   

We set out to create layered structures, expecting to obtain OD polytypes due to different local layer symmetry. We were indeed successful with the systematic twins oxESEM (**3*a***) and ASYM (**6**). Surprisingly though, we observed numerous other crystallographic phenomena, which are not caused by local layer symmetry but by the remarkable flexibility of the layer contacts. On the one hand, a given layer contact can accommodate significant distortion leading to the incommensurate modulation of ESEM (**3**). On the other hand, identical layers can connect in fundamentally different ways as observed in the non-OD polytypism of TSEM (**2**) and DSEM (**4**) and the layer contacts can accomodate the different molecular arrangements observed in the temperature-dependent polymorphism of oxBSEM (**1*b***).

These phenomena demonstrate the necessity of a generalization of space-group symmetry to local symmetry. These so-far unexplored groupoids are necessary not only for the description of polytypism, but also of structural relationships like the merotypism of BSEM (**1**) and TSEM (**2**).

Future synthetic work will focus on the core of the layers (alterations of the aromatic spacer and an elongation of the methylthio groups), as well as the application of other silyl groups like TBDMS or TIPS to induce different layer contacts as in NSEM-TBDMS (**5**). Besides studying crystallographic phenomena, the application of acene or polythiophene derivatives may also enable the application of the layer motifs in the field of functional organic materials. To achieve this goal an adjustment of the steric bulk of the cores and the size of the silyl groups will be needed to obtain an optimum face-to-face stacking of the extended aromatic cores. Moreover, the merotypism of BSEM (**1**) and TSEM (**2**) presents an opportunity for the controlled epitaxial growth of different kinds of molecules, a crucial point in the design of devices.

## Supplementary Material

Crystal structure: contains datablock(s) ASYM, BSEM, BSEMOX1, BSEMOX2, BSEMOX3, DSEM1, DSEM2, ESEM, ESEMOX, NSEM, TSEM. DOI: 10.1107/S2052252515011665/yc5004sup1.cif

Structure factors: contains datablock(s) ASYM. DOI: 10.1107/S2052252515011665/yc5004ASYMsup2.hkl

Structure factors: contains datablock(s) BSEM. DOI: 10.1107/S2052252515011665/yc5004BSEMsup3.hkl

Structure factors: contains datablock(s) BSEMOX1. DOI: 10.1107/S2052252515011665/yc5004BSEMOX1sup4.hkl

Structure factors: contains datablock(s) BSEMOX2. DOI: 10.1107/S2052252515011665/yc5004BSEMOX2sup5.hkl

Structure factors: contains datablock(s) BSEMOX3. DOI: 10.1107/S2052252515011665/yc5004BSEMOX3sup6.hkl

Structure factors: contains datablock(s) DSEM1. DOI: 10.1107/S2052252515011665/yc5004DSEM1sup7.hkl

Structure factors: contains datablock(s) DSEM2. DOI: 10.1107/S2052252515011665/yc5004DSEM2sup8.hkl

Structure factors: contains datablock(s) ESEMOX. DOI: 10.1107/S2052252515011665/yc5004ESEMOXsup9.hkl

Structure factors: contains datablock(s) NSEM. DOI: 10.1107/S2052252515011665/yc5004NSEMsup10.hkl

Structure factors: contains datablock(s) TSEM. DOI: 10.1107/S2052252515011665/yc5004TSEMsup11.hkl

Supporting information file. DOI: 10.1107/S2052252515011665/yc5004sup12.pdf

Structure factors: contains datablock(s) ESEM. DOI: 10.1107/S2052252515011665/yc5004ESEMsup13.hkl

CCDC references: 927469, 927470, 927472, 927473, 928106, 928107, 928108, 928110, 1407074, 1407075, 1407076

## Figures and Tables

**Table 1:** Torsion angles in the title compounds () For structures with two crystallographically different molecules each molecule is listed in a separate row. For the incommensurately modulated ESEM (**3**) minimum and maximum values are indicated. For thiophene spacers the S atom was used as terminal atom, for benzene spacers the C atom with a torsion angle >90. For the CSi(CC)CC torsion angle the atom with the angle closest to 0 or 180 was chosen.

Molecule	CCspacer	CC(spacer)CC	CCSCH_3_	CSi(CC)CC
BSEM	144.26(8)	180	138.00(6)	30.09(8)
TSEM	152.0(3)	68.5(5)	129.3(3)	28.8(4)
ESEM	156.36165.31(12)	47.452.4(3)	149.81162.53(17)	149.81162.53(17)
DSEM, polytype I	156.66(19), 163.93(17)	50.2(3)	121.71(19), 111.17(19)	160.0(2), 158.9(2)
DSEM, polytype II	157.1(4), 162.7(4)	51.4(7)	124.7(4), 111.2(5)	160.8(4), 22.1(5)
oxBSEM, polymorph I	132.80(11)	180	57.32(11)	151.46(10)
	131.49(10)	180	62.46(9)	24.84(10)
oxBSEM, polymorph II	127.7(4)	180	71.8(4)	176.7(5)
	128.5(4)	180	109.2(4)	29.9(3)
oxBSEM, polymorph III	124.93(17)	180	84.82(15)	23.9(18)
oxESEM	44.6(3), 63.7(3)	14.9(3)	80.0(2), 66.8(2)	159.6(2), 151.0(2)
	46.2(3), 64.2(3)	17.2(3)	78.8(2), 67.8(3)	159.6(2), 153.4(2)
NSEM-TBDMS		76.32(12)	179.50(7), 164.04(8)	170.82(7), 10.18(8)
ASYM			179.3(2)	166.7(2), 178.4(2)

**Table 2:** Symmetry of the overall structures and the molecular layers and operations relating adjacent layers in the crystals under investigation For the incommensurately modulated ESEM (**3**), symmetry and operations of the basic structure are listed. Pseudosymmetry of OD structures is not listed.

Structure	Space group	Layer group	Operations relating adjacent layers
BSEM	*C*2/*c*	*p*(1)2/*c*1	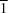 , 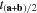 , 2_1_ [010], *n* [010]
TSEM	*I*2/*c*	*p*(1)2/*c*1	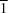 , 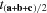 , 2_1_ [010], *a* [010]
ESEM, basic structure	*I*2/*c*	*p*(1)2/*c*1	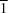 , 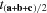 , 2_1_ [010], *a* [010]
DSEM, polytype I	*C*2/*c*	*p*(1)2_1_/*c*1	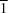 , 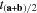 , 2_1_ [010], *n* [010]
DSEM, polytype II	*Pccn*	*p*(1)2_1_/*c*1	2_1_ [100], *c* [100], 2 [001], *n* [001]
oxBSEM, polymorph I	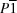	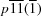	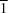 , *t*_**c**_
oxBSEM, polymorph II	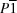	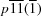	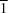 , *t*_**c**_
oxESEM	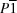	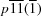	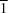 , *t*_**c**_
NSEM-TBDMS	*P*2_1_2_1_2_1_	*p*12_1_(1)	2_1_ [100], 2_1_ [001]
ASYM	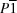	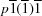	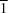 , *t*_**b**_

**Table 3:** Prominent intermolecular contacts in the *d*_i_/*d*_e_ fingerprint plots of the I and II polymorphs of oxBSEM (**1*b***) marked in Fig. 23 ▸

	Polymorph I		Polymorph II	
Region	Atoms	Molecular groups	Atoms	Molecular groups
1	C5H5O2	enesulfone		
2	C11H112C7	Methylsulfoneyne	C11H113C3	Methylsulfonebenzene
3	C11H111C3	Methylsufonebenzene	C11H112C2	Methylsulfonebenzene
4	C9H93O2	TMSsulfone	C5H5O2	Enesulfone
5			C5H5O1	Enesulfone
6	C3H3H3C3	Benzenebenzene		
