# Supplementary material for: Crystal chemistry of layered structures formed by linear rigid silyl-capped molecules
Source: IUCrJ. 2015 Aug 20;2(Pt 5):584–600. doi: 10.1107/S2052252515011665 (PMC4547826; doi:10.1107/S2052252515011665)
Supplement: Supplementary file 12 [file m-02-00584-sup12.pdf]

IUCrJ

Volume 2 (2015)

Supporting information for article:

Crystal chemistry of layered structures formed by linear rigid silyl-capped molecules

Daniel Lumpi, Paul Kautny, Berthold Stöger and Johannes Fröhlich

Crystal chemistry of layered structures formed by linear rigid silyl-capped molecules - Experimental details

Daniel Lumpi,[a] Paul Kautny,[a] Berthold Stöger[b]* and Johannes Fröhlich[a]

[a]*Institute of Applied Synthetic Chemistry, Vienna University of Technology, Vienna Austria, and* [b]*Institute of Chemical Technologies and Analytics, Vienna University of Technology, Vienna Austria. E-mail: bstoeger@mail.tuwien.ac.at*

1. Single crystal diffraction

Crystals of the title compounds were embedded in perfluorinated oil and attached to a thin glass fiber. Intensity data were collected using Mo$K\overline{\alpha}$ radiation ($\lambda = 0.71073$ Å) on a Bruker Kappa APEX II diffractometer with κ-geometry and a 0.7 mm collimator. Due to weak diffraction, notably of platy crystals, in some cases crystals of a size slightly exceeding the beam diameter were used to maximize intensities. It has been shown that this generally does not affect the refinement quality (Tan & Ng, 2014). Full spheres were collected in fine-sliced ω- and ϕ-scans up to a 2θ angle where reflections were still visible in preliminary scans, with the exception of polymorph III of oxB-SEM, which was only measured up to $2\theta = 27.6°$, albeit scattering to higher angles. In general frame data were reduced to intensity values with SAINT-Plus and corrected for absorption effects using the multi-scan approach implemented in SADABS or TWINABS (Bruker, 2008). The structures were solved by charge-flipping implemented in SUPERFLIP (Palatinus & Chapuis, 2007) and refined against F values

with JANA2006 (Petříček *et al.*, 2014). Non-H atoms were refined with anisotropic ADPs. H atoms were placed at computed positions and refined as riding on the parent C-atoms. Details of data collections and structure refinements are compiled in Tables 1–4. Deviations from the standard procedure and specific remarks will be given in the following sections.

Table 1. *Details on the crystal structure determinations of the methylthio compounds BSEM (**1**), TSEM (**2**), and both of DSEM (**4**).*

	BSEM	TSEM	DSEM, polytype I	DSEM, polytype II
formula	$C_{22}H_{30}S_2Si_2$	$C_{20}H_{28}S_3Si_2$	$C_{22}H_{32}O_2S_3Si_2$	$C_{22}H_{32}O_2S_3Si_2$
molecular weight	414.8	420.8	480.8	480.8
crystal color	clear yellow	clear yellow	clear yellow	clear yellow
crystal habit	plate	fragment	rhombic prism	plate
crystal size [mm^3]	0.77×0.25×0.03	0.09×0.07×0.01	0.87×0.66×0.03	0.60×0.51×0.01
temperature [K]	100	100	100	100
space group	$C2/c$	$I2/c$	$C2/c$	$Pccn$
a [Å]	34.148(6)	34.443(3)	34.344(2)	33.630(10)
b [Å]	6.8690(12)	6.7415(4)	8.1665(5)	8.271(2)
c [Å]	10.3442(18)	10.1978(8)	20.0791(12)	19.717(6)
α [°]	90	90	90	90
β [°]	98.343(8)	96.889(5)	100.532(2)	90
γ [°]	90	90	90	90
V [Å^3]	2400.7(7)	2350.8(3)	5536.7(6)	5484(3)
Z	4	4	8	8
Z'	$\frac{1}{2}$	$\frac{1}{2}$	1	1
density [g cm^{-3}]	1.147	1.189	1.153	1.164
θ range [°]	1.2–35.0	1.2–30.2	1.2–27.6	1.2–25.1
μ [mm^{-1}]	0.326	0.419	0.369	0.373
Trans. coeff. $T_{\min}$, $T_{\max}$	0.91, 0.99	0.97, 1.00	0.74, 0.99	0.90, 1.00
reflections total	38270	29932	77428	48785
reflections unique	5255	3045	6397	4671
reflections obs. [$I > 3\sigma I$]	4193	2366	4903	3185
parameters	118	115	262	262
R_{int}	0.0347	0.0643	0.0380	0.0792
h	-54→54	-45→48	-44→44	-9→9
k	-11→11	-9→9	-10→10	-13→13
l	-16→16	-14→14	-26→26	-17→17
$\Delta\rho$max [e Å^{-3}]	0.43	1.53	0.87	0.75
$\Delta\rho$min [e Å^{-3}]	-0.22	-1.41	-0.75	-0.74
GooF	2.19	2.56	3.02	3.22
R_{obs}	0.0307	0.0780	0.0489	0.0869
wR_{all}	0.0442	0.0625	0.0588	0.0848
twin operation	-	twofold rotation about [001]	-	-
twin volume fraction	-	50.39:49.61(17)	-	-

Table 2. *Details on the crystal structure determinations of the methylsulfonyl compounds oxBSEM (**1**) (polymorphs I, II and III) and oxESEM (**3b**).*

	oxBSEM, I	oxBSEM, II	oxBSEM, III	oxESEM
formula	$C_{22}H_{30}O_4S_2Si_2$	$C_{22}H_{30}O_4S_2Si_2$	$C_{22}H_{30}O_4S_2Si_2$	$C_{22}H_{30}O_8S_3Si_2$
molecular weight	478.8	478.8	478.8	574.8
crystal color	clear colorless	clear colorless	clear colorless	clear yellow
crystal habit	plate	plate	plate	block
crystal size [mm^3]	0.70×0.65×0.10	0.70×0.65×0.10	0.45×0.15×0.03	0.63×0.45×0.28
temperature [K]	150	100	100	100
space group	$P\bar{1}$	$P\bar{1}$	$P\bar{1}$	$P\bar{1}$
a [Å]	6.8197(3)	7.3096(2)	5.7300(3)	10.5399(4)
b [Å]	12.1073(5)	11.3935(3)	10.2961(5)	13.8276(5)
c [Å]	16.1123(7)	18.7425(6)	10.9801(6)	19.9987(8)
α [°]	92.3607(19)	73.167(2)	85.9433(17)	79.940(2)
β [°]	93.9781(19)	105.319(2)	79.7181(16)	84.589(2)
γ [°]	98.3425(19)	118.926(2)	80.1150(15)	89.771(2)
V [Å^3]	1311.34(10)	1293.21(7)	627.39(6)	2856.81(19)
Z	2	2	1	4
Z'	$\frac{2}{2}$	$\frac{2}{2}$	$\frac{1}{2}$	2
density [g cm^{-3}]	1.212	1.229	1.267	1.336
θ range [°]	1.3–35.1	1.15–30.15	1.89–27.6	1.0–30.1
μ [mm^{-1}]	0.318	0.323	0.332	0.385
Trans. coeff. $T_{\min}$, $T_{\max}$	0.81,0.97	0.81,0.97	0.89,0.98	0.81,0.90
reflections total	57779	40880	16087	57022
reflections unique	11485	7541	2909	13689
reflections obs. [$I > 3\sigma I$]	8331	5106	2371	11291
parameters	271	271	136	632
$R_{\rm int}$	0.0297	0.0625	0.0237	0.0348
h	-9→11	-10→10	-6→7	-14→14
k	-19→19	-15→16	-13→13	-19→19
l	-26→25	-26→26	-14→14	-28→28
$\Delta\rho$max [e Å^{-3}]	0.44	1.17	0.31	0.45
$\Delta\rho$min [e Å^{-3}]	-0.39	-0.70	-0.26	-0.60
GooF	2.15	3.05	2.19	1.96
$R_{\rm obs}$	0.0350	0.0740	0.0300	0.0372
$wR_{\rm all}$	0.0443	0.0925	0.0393	0.0422
twin operation	-	-	-	twofold rotation about [100]
twin volume fraction	-	-	-	60.92:39.08(7)

Table 3. *Details on the crystal structure determinations of the non-spacer extended compounds NSEM-TBDM (**5**) and ASYM (**6**)*

	NSEM-TBDMS	ASYM
formula	$C_{22}H_{38}S_2Si_2$	$C_{13}H_{22}S_1Si_2$
molecular weight	422.8	266.6
crystal color	clear colorless	clear colorless
crystal habit	block	block
crystal size [mm^3]	0.70×0.56×0.50	0.82×0.61×0.20
temperature [K]	100	100
space group	$P2_12_12_1$	$P\overline{1}$
a [Å]	7.7849(4)	7.2016(4)
b [Å]	10.5146(5)	9.7603(4)
c [Å]	31.2712(14)	12.2829(4)
α [°]	90	95.4952(16)
β [°]	90	92.510(2)
γ [°]	90	101.346(2)
V [Å^3]	2559.7(2)	840.86(6)
Z	4	2
Z'	1	1
density [g cm^{-3}]	1.097	1.052
θ range [°]	2.04–35.05	1.7–30.2
μ [mm^{-1}]	0.306	0.313
Trans. coeff. $T_{\min}$, $T_{\max}$	0.78, 0.81	0.80, 0.94
reflections total	101389	21588
reflections unique	11125	4622
reflections obs. [$I > 3\sigma I$]	10833	3651
parameters	236	146
R_{int}	0.0238	0.0345
h	-12→11	-9→9
k	-16→16	-13→13
l	-48→50	-17→17
$\Delta\rho$max [e Å^{-3}]	0.33	0.61
$\Delta\rho$min [e Å^{-3}]	-0.28	-0.39
GooF	2.72	2.27
R_{obs}	0.0267	0.0524
wR_{all}	0.0400	0.0549
Flack parameter	0.03(3)	-
twin operation	-	twofold rotation about [100]
twin volume fraction	-	83.78:16.22(11)

Table 4. *Details on the crystal structure determinations of the incommensurately modulated structure ESEM (**3**).*

	ESEM
formula	$C_{22}H_{30}O_2S_3Si_2$
molecular weight	478.8
crystal color	clear yellow
crystal habit	plate
crystal size [mm^3]	0.62×0.23×0.02
temperature [K]	100
superspace group	$I2/c(0\sigma_2 0)s0$
a [Å]	32.4653(8)
b [Å]	8.4003(5)
c [Å]	10.0737(3)
β [°]	102.301(2)
V [Å^3]	2684.20(19)
$\mathbf{q}$	0.6223(1)$\mathbf{b}$
Z	4
θ range [°]	1.8–27.5
μ [mm^{-1}]	0.38
unique reflections (all, obs)	15382, 9184
unique main reflections (all, obs)	3080, 2307
unique first order sat (all, obs)	6156, 4248
unique second order sat (all, obs)	6146, 2629
observation criterion	$I > 3\sigma I$
parameters	510
R_{int}	0.0672
h	-41→41
k	-11→11
l	-13→12
m	-2→2
GooF	2.36
$R_{\mathrm{obs}}/wR_{\mathrm{all}}$	
all reflections	6.28/6.69
main reflections	5.87/6.47
first order satellites	4.78/4.91
second order satellites	11.35/12.71

1.1. *Twinning of TSEM (2), oxESEM (3b) and ASYM (6).*

For TSEM (**2**), oxESEM (**3b**) and ASYM (**6**), automatic unit cell determination failed at determining reasonable lattice parameters. Therefore the locations of the diffraction spots in reciprocal space were analyzed manually using RLATT (Bruker, 2008). In all three cases the spots could be assigned to two domains, related by rotation

of 180° about [001] (TSEM (**2**)) and [100] (oxESEM (**3b**), ASYM (**6**)). Intensity data of the twin domains were integrated concurrently and written to "HKLF5" files with overlap information.

1.2. TSEM (2).

TSEM (**2**) was refined in the non-standard space group setting $I2/c$ (standard setting $C2/c$) to highlight the close crystallographic relationship with BSEM (**1**). The standard setting is related to the chosen one by

$$(\mathbf{a}_{\rm std}, \mathbf{b}_{\rm std}, \mathbf{c}_{\rm std}) = (\mathbf{a}, \mathbf{b}, \mathbf{c}) \begin{pmatrix} 1 & 0 & 0 \\ 0 & \bar{1} & 0 \\ 1 & 0 & \bar{1} \end{pmatrix}.$$

Due to the small size, the crystal was only weakly diffracting, leading to mediocre residuals and large peaks in the difference Fourier density.

1.3. DSEM (4).

The two polytypes of DSEM (**4**) were isolated from the same crystallization vessel. Polytype I grew on the walls and featured satisfactory diffraction quality. Polytype II was isolated from the bottom of the wessel among oil and featured weak diffraction intensities and diffuse scattering. Accordingly, the residuals were comparatively large.

*1.4. oxBSEM (**1b**).*

When mounting samples of oxBSEM (**1b**) on the diffractometer cooled in a stream of N_2 to our routine measurement temperature of 100 K, the crystals burst suggesting a phase transition. Therefore we slowly (2 K/min) cooled a crystal from room temperature to 150 K, while monitoring the lattice parameters. Since no phase transition was observed down to 150 K, a data set was collected at this temperature. On further cooling, a phase transition was apparent by fragmentation of the crystal accompanied by a dramatic worsening of reflection quality. Despite mediocre data quality we were

able to solve and refine the structure, though with high residuals and large peaks in the differene Fourier density.

To highlight the structural relationship between polymorphs I and II of oxBSEM (**1b**), the latter was refined using a non-reduced setting. The reduced setting is related to the chosen one by

$$(\mathbf{a}_{\text{red}}, \mathbf{b}_{\text{red}}, \mathbf{c}_{\text{red}}) = (\mathbf{a}, \mathbf{b}, \mathbf{c}) \begin{pmatrix} 1 & 1 & 1 \\ 0 & 1 & 0 \\ 0 & 0 & 1 \end{pmatrix}.$$

*1.5. ESEM (**3**).*

Since automatic indexing of the reflections failed, the locations of the diffraction spots in reciprocal space were analyzed manually using RLATT (Bruker, 2008). The strongest spots could be indexed using a C-centered monoclinic lattice. The remaining diffraction spots were interpreted as satellites located at $\pm\sigma_2\mathbf{b}^*$ and $\pm2\sigma_2\mathbf{b}^*$ from main reflections, with irrational σ_2 close to $\frac{5}{8}$.

Data reduction turned out to be difficult, since first and second order satellites were close and reflections featured distinct enlargement in $\mathbf{a}^*$ direction. The best overall result was obtained using EVAL14 (Duisenberg *et al.*, 2003), by tuning anisotropic mosaicity. Nevertheless, intensities of the strongest reflections were systematically overestimated, leading to slightly worse partial reliability factors of main reflections compared to first order satellites.

Systematic absences of the main reflections indicated a superspace group derived from Ic or $I2/c$. Since satellites $0k0m$ with $|m| = 1$ were absent, a structure solution in superspace group $I2/c(0\sigma_2 0)s0$ was attempted. The correctness of the choice was confirmed by the symmetry of the four-dimensional electron density obtained by charge-flipping as implemented in SUPERFLIP (Palatinus & Chapuis, 2007). The positions of all non-H atoms and first order displacive modulation functions were

directly located in SUPERFLIP output. To achieve decent reliability factors, notably concerning satellites, the positions of the non-H atoms were refined with second order harmonics. The ADPs of the heavy atoms S and Si were modulated with second order harmonics, for C and O atoms with first order harmonics. H atoms were placed at computed positions and refined as riding on the parent C-atoms. The methyl groups were fixed into *anti*-positions to obtain converging refinements.

The C9 atom of the TMS group features large ADPs in parts of internal space hinting towards disorder. Introducing discontinuous modulation functions for C9 (Legendre polynomials) led to distinctly more reasonable ADPs. Unfortunately attachment of H atoms to this C9 atom resulted in non-converging refinements and thus the continuous harmonics were used in the final refinements.

References

Bruker Analytical X-ray Instruments, Inc., Madison, WI, USA, (2008). APEX2, RLATT, SAINT, SADABS and TWINABS.

Duisenberg, A. J. M., Kroon-Batenburg, L. M. J. & Schreurs, A. M. M. (2003). *Appl. Cryst.* **36**, 220–229.

Palatinus, L. & Chapuis, G. (2007). *J. Appl. Cryst.* **40**, 786–790.

Petříček, V., Dušek, M. & Palatinus, L. (2014). *Z. Kristallogr.* **229**, 345–352.

Tan, S. L. & Ng, S. W. (2014). *J. Appl. Cryst.* **47**, 1443–1444.
